# Decoding PVAT Complexity in Vascular Remodeling: Multimodal Single-Cell Technologies Unveil Novel Therapeutic Targets

**DOI:** 10.3390/cells15181645

**Published:** 2026-09-11

**Authors:** Yujun Xu, Matthew R. Bersi

**Affiliations:** Department of Mechanical Engineering & Materials Science, Washington University in St. Louis, St. Louis, MO 63130, USA; x.yujun@wustl.edu

**Keywords:** perivascular adipose tissue (PVAT), single-cell transcriptomics (scRNA-seq), vascular mechanics, cellular plasticity, intercellular crosstalk

## Abstract

Perivascular adipose tissue (PVAT) has emerged as a central regulator of vascular homeostasis in large arteries, with its dysfunction driving the pathogenesis of atherosclerosis, hypertension, and metabolic diseases. In the past, our understanding of PVAT was limited by bulk omics approaches that obscured the tissue’s profound spatial, cellular, and functional heterogeneity. Here, we review how multimodal single-cell technologies, including single-cell RNA sequencing, spatial transcriptomics, and AI integration, are now decoding the PVAT ecosystem with unprecedented resolution. These approaches have generated an integrated atlas that reveals depot-specific cellular architectures, dynamic phenotypic plasticity, and spatially organized crosstalk between biological circuits that drive vasculopathy. We highlight novel biomarkers and actionable therapies emerging from these insights, such as *Dpp4^+^* preadipocytes and BMP4-induced browning. Finally, we propose a translational roadmap that prioritizes human PVAT biobanking, CRISPR-based lineage tracing, and AI-driven modeling to transform these mechanistic discoveries into precision interventions. Ultimately, by establishing PVAT as a central orchestrator of vascular homeostasis, these single-cell insights provide the mechanistic foundation to therapeutically intercept cardiometabolic disease at its cellular source.

## 1. Introduction

Perivascular adipose tissue (PVAT) is no longer considered to be an inert structural support surrounding the vasculature, but is rather known to be a dynamic endocrine and paracrine organ indispensable for vascular homeostasis [1,2,3]. Recent investigations have revealed the role of PVAT in regulating vascular tone [4], inflammation, and extracellular matrix (ECM) remodeling through bidirectional communication with the vascular wall [5,6,7,8,9,10,11]. Its anatomical adjacency creates a specialized niche where paracrine signaling, immune modulation, thermogenic activity, and mechanotransduction enable dynamic crosstalk between endothelial cells (ECs), vascular smooth muscle cells (VSMCs), and resident immune populations [12,13]. This functional interdependence establishes PVAT as an integral component of the vascular unit, effectively constituting a “fourth layer” of the vessel wall [14,15,16].

Because of its unique physical and functional integration with the vessel wall, PVAT exhibits a critical duality in the context of cardiovascular physiology. Under homeostatic conditions, PVAT exerts a net protective influence by secreting vasodilatory and anti-inflammatory adipokines, such as adiponectin and nitric oxide, that modulate vascular tone, suppress VSMC proliferation, and preserve endothelial integrity [3,17]. However, in prevalent cardiometabolic pathologies including obesity, metabolic syndrome, and diabetes, PVAT undergoes maladaptive remodeling [18,19] characterized by adipocyte hypertrophy, mitochondrial dysfunction, a shift toward secretion of pro-inflammatory mediators (e.g., leptin, resistin, IL-6, MCP-1), and robust immune cell infiltration [20,21]. Consequently, this transformation converts PVAT from its baseline vasoprotective role to a more pathogenic role that drives endothelial dysfunction, aberrant VSMC activation, and ECM dysregulation [18,22]. Such paradoxical positioning renders PVAT as both a therapeutic target and biomarker reservoir for a spectrum of cardiovascular diseases, from atherosclerosis and hypertension to aortic aneurysms [23,24]. Despite its potential clinical utility, the precise cellular and molecular mechanisms orchestrating this functional switch in PVAT maladaptation remain elusive.

Conventional bulk transcriptomics, proteomics, and histomorphometry have advanced our fundamental understanding of PVAT’s cellular and ECM structure, endocrine functions, and macroscopic contributions to vascular disease. However, these bulk technologies produce averaged signals across heterogeneous cellular compartments that effectively mask spatial patterning, transient cell states, and adipose depot-specific biology [25,26]. In the aorta, these measurement limitations have prevented resolution of functional distinctions between thermoprotective thoracic PVAT (T-PVAT; above the diaphragm) and inflammation-prone abdominal PVAT (A-PVAT; below the diaphragm). Consequently, there are fundamental questions related to PVAT function and adaptation that have persisted: Which cellular subpopulations initiate PVAT dysfunction? How do spatial interactions within PVAT mechanistically propagate vasculopathy? And what molecular switches govern phenotypic transitions of PVAT?

The recent emergence of multimodal single-cell technologies has the potential to address these critical research gaps, thereby catalyzing a new paradigm of PVAT biology that positions this tissue as a central processor of cardiovascular health and disease. For instance, single-cell RNA sequencing (scRNA-seq) profiles transcriptomes from individual cells dissociated from whole tissues to enable the precise identification of distinct cell types and disease-associated cell states [27]. Single-nucleus RNA sequencing (snRNA-seq) provides a complementary technique which isolates RNA from nuclei rather than cells and is particularly important for difficult-to-dissociate tissues, frozen tissues, and lipid-rich adipocytes [28,29]. Spatial transcriptomic approaches further preserve anatomical information by mapping gene-expression patterns from local transcriptomic analysis to defined locations within intact tissue sections. This approach enables the investigation of spatially organized cellular compositions and signaling relationships within tissues of interest [30]. Additional multimodal approaches, such as assay for transpose-accessible chromatin using sequencing (ATAC-seq) and cellular indexing of transcriptomes and epitopes by sequencing (CITE-seq), integrate transcriptional information with chromatin accessibility or surface protein expression, respectively, to provide a comprehensive view of epigenetic changes and cellular regulation [29,31]. Together, these technologies generate cell-resolved and spatially contextualized information that is fundamentally inaccessible through bulk averaging approaches alone. Here, we synthesize recent findings on how single-cell technology has decoded the exceptional cellular diversity and plasticity of PVAT, mapped the pathogenic communication circuits in PVAT driving vasculopathy, and unveiled a potential pipeline of novel PVAT-specific biomarkers and therapeutic targets. Finally, we culminate by positing a forward-looking roadmap intended to overcome persistent challenges in human validation, dynamic imaging, and species-specific modeling, with the goal of charting a course toward establishing PVAT as a therapeutically targetable regulator of cardiovascular health and disease. While the aorta-PVAT axis serves as the foundational focus of this review, dedicated single-cell transcriptomics studies specific to aortic PVAT remain relatively limited. We have therefore incorporated key findings from coronary PVAT and systemic adipose biology, as these diverse perivascular beds share highly conserved immunometabolic signaling mechanisms and can inform our broader understanding of PVAT and vascular pathophysiology.

To construct this narrative review, a series of literature searches were conducted in PubMed and relevant NCBI databases. A primary focus was placed on reviewing recent articles published between 2018 and 2026 in an effort to capture the rapid emergence and maturation of single-cell technologies. Principal literature search keywords included combinations of “perivascular adipose tissue,” “PVAT,” “single-cell RNA sequencing,” “scRNA-seq,” “spatial transcriptomics,” and “vascular remodeling”. Studies were prioritized for inclusion in the current review if they utilized single-cell or high-resolution spatial omics technologies and were able to identify novel cellular subpopulations, state transitions, or intercellular communication networks within PVAT that were previously undetectable by bulk methodologies.

## 2. Decoding Cellular Diversity Through Single-Cell Atlases

By applying the single-cell- and spatially resolved technologies described above, researchers are now able to overcome the methodological barriers of bulk omics approaches, which average signals across heterogeneous compartments and effectively obscure the spatial, functional, and depot-specific biology of PVAT [25,26,32]. For example, single-cell analysis revealed a mischaracterization of macrophage activation states in PVAT identified from bulk transcriptomics [33,34]; while bulk approaches identified a general macrophage activation, single-cell analysis uncovered distinct M1, M2, lipid-associated macrophages (LAM), and metabolically activated macrophage (MMe) populations with specific functions related to PVAT biology [33,35,36]. Similarly, adipocyte heterogeneity was largely obscured by bulk approaches, yet single-cell analysis of PVAT revealed the presence of multiple adipocyte subtypes with different metabolic profiles and disease associations [25,37]. Indeed, comprehensive single-cell atlases integrating over 400,000 cells across multiple PVAT depots and disease states have identified more than sixty transcriptionally distinct cellular subpopulations within PVAT, organized into four principal compartments (e.g., adipocyte, immune, stromal, and vascular), that exhibit specialized functions and spatial organization [25,32,38].

**Figure 1:**
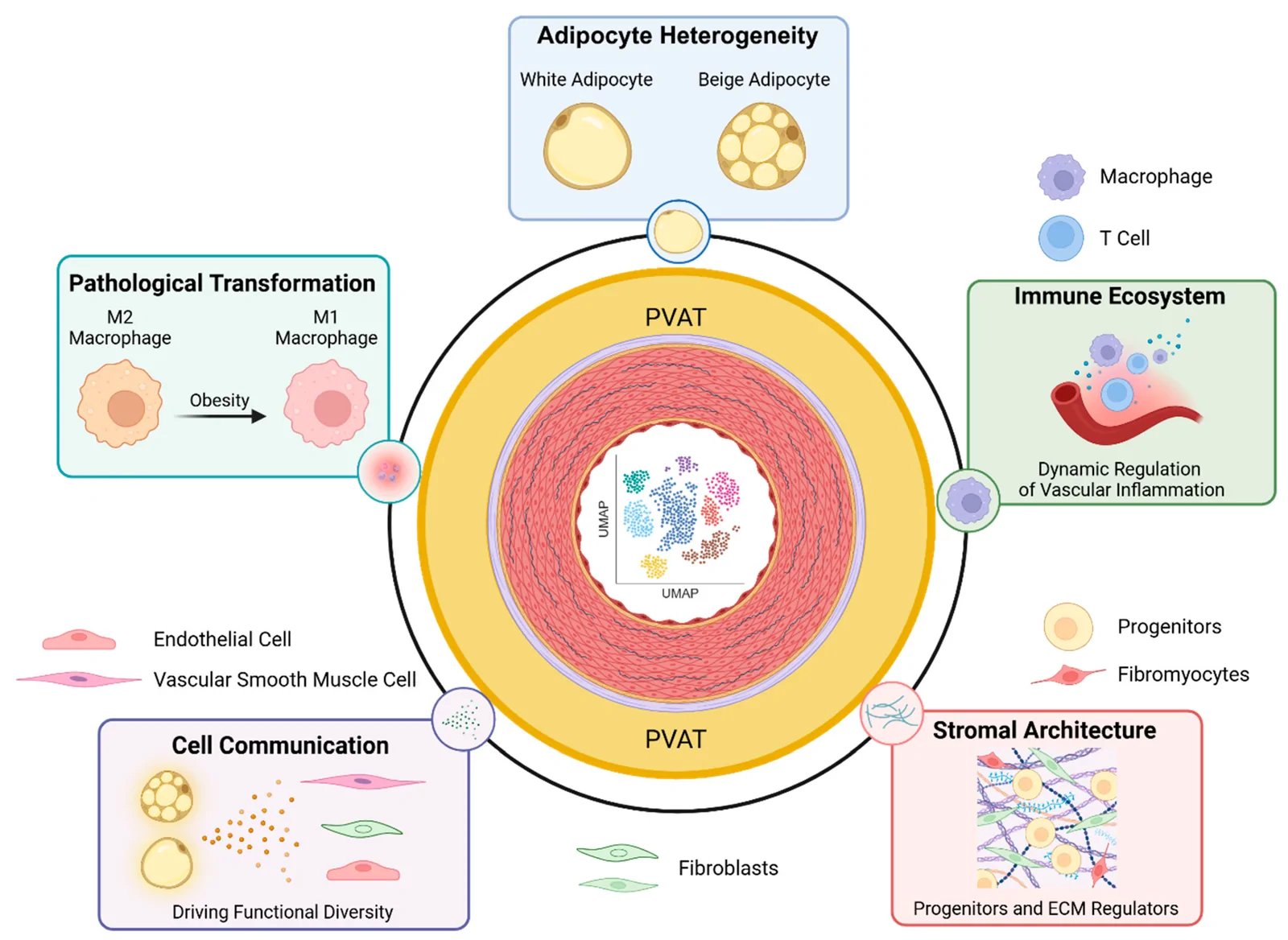
Multimodal single-cell atlases decode the cellular complexity and pathological plasticity of PVAT. The central schematic illustrates the paradigm shift driven by single-cell transcriptomics (represented by the central UMAP visualization within the vascular lumen). This technology deconstructs the bulk PVAT into a highly organized, multi-cellular ecosystem surrounding the vessel wall, comprising distinct adipocyte, immune, stromal, and vascular cell lineages. High-resolution mapping reveals several core biological dimensions of PVAT: (**Top**) Adipocyte Heterogeneity: identification of functionally distinct adipocyte subpopulations, spanning from thermogenic beige to lipid-storing white phenotypes, which dictate local PVAT metabolism. (**Right**) Immune Ecosystem: a dynamic network of resident and recruited immune cells, including macrophages and T cells, that actively surveil the perivascular niche to regulate vascular inflammation. (**Bottom Right**) Stromal Architecture: a specialized structural compartment comprising adipogenic progenitor cells and matrix-producing fibromyocytes that govern ECM mechanics and vascular stiffness. (**Bottom Left**) Cell Communication: spatially coordinated intercellular signaling networks connecting adipocytes, stromal cells, and PVAT immune populations with the resident vascular wall cells (e.g., endothelial and vascular smooth muscle cells) drive depot-specific functional diversity. (**Left**) Pathological Transformation: chronic metabolic stressors, such as obesity, catalyze a fundamental phenotypic reorganization within PVAT, exemplified by polarization from a vasoprotective M2 to pro-inflammatory M1 macrophage states, potentiating a pathogenic cycle of vascular remodeling.

Inclusion of spatial transcriptomics preserves the anatomical context alongside the transcriptional profile for each measurement in order to illuminate zonal organization within tissues and map ligand-receptor interactions at PVAT-vascular interfaces [38,39]. Other spatial approaches, such as spatial metabolomics, have identified features of inflamed adipocytes, such as ceramide gradients, in cardiometabolic disorders including diabetes [40,41]. Adoption of artificial intelligence (AI) analysis frameworks can reliably synthesize such multimodal data streams and has been leveraged to predict inflammatory hotspots within PVAT from cellular colocalization patterns in spatial datasets [29]. As a step further, multimodal integration-combining transcriptomics, chromatin accessibility (snATAC-seq), histone modifications (snCUT&Tag), and surface proteomics (CITE-seq)—has decoded gene-regulatory networks driving pathological PVAT reprogramming [29,31]. By resolving the network of PVAT cellular diversity at such unprecedented resolution, these approaches have transformed our understanding of PVAT from a simple adipose support layer into a dynamic and structured cellular ecosystem wherein specialized cells engage in spatially orchestrated crosstalk with one another and the adjacent vasculature [25,26,28,31]. These findings have redefined our understanding of adipose tissue biology and identified new PVAT-specific therapeutic targets that were previously unrecognized (Figure 1).

### 2.1. Comprehensive Cellular Mapping Reveals Unprecedented Heterogeneity

PVAT constitutes a microcosm of cellular heterogeneity whereby the functional specialization of the cellular compartment dictates interactions with cells and neighboring tissues. Development of a single-cell atlas for PVAT demonstrated that the adipocyte compartment exhibits remarkable functional diversification, with five evolutionarily conserved subtypes distributed throughout aortic PVAT depots. In particular, (1) thermogenic adipocyte progenitors (TAPs), localized predominantly in T-PVAT and characterized by *Pdgfrα^+^/Cd34^+^/Prdm16^+^* expression [8,42], generate beige adipocytes through BMP4-mediated mitochondrial biogenesis [43]. Single-cell pseudotime analysis demonstrates the differentiation of TAPs along a UCP1-high trajectory driven by adrenergic signaling and cold exposure [8,19]; note that uncoupling protein 1 (UCP1) is a canonical marker of brown adipocytes and a hallmark of thermogenic adipose identity [44]. Functionally, TAP-derived beige adipocytes secrete protective adipokines (e.g., adiponectin, apelin) and catabolize circulating lipids, thereby reducing perivascular lipid deposition and oxidative stress [8]. Moreover, TAP abundance inversely correlates with atherosclerotic severity in human cohorts [23,45]. On the other hand, (2) lipid-storing inflammatory adipocytes (LSIAs) are predominantly expressed in A-PVAT during obesity and exhibit hypertrophic morphology and excess accumulation of ceramides that trigger NLRP3 inflammasome activation [6]. LSIAs secrete interleukin-6 (IL-6) and monocyte chemoattractant protein-1 (MCP-1), effectively driving endothelial dysfunction through impaired insulin signaling and endoplasmic reticulum stress [35]. These cells characteristically exhibit stearoyl-CoA desaturase 1 (*Scd1*) mRNA-protein discordance, which leads to impaired lipid desaturation and promotes ceramide accumulation [41]. A third population of adipocytes, (3) fibro-inflammatory adipocytes (FIAs), resides near fibrotic zones in aneurysmal PVAT and sclerotic vessels and uniquely co-expresses adipocyte (*Adipoq*, *Fabp4*) and fibroblast (*Col1a1*, *Col3a1*, *Acta2*) genetic markers, suggesting potential transdifferentiation between cell types [46]. Spatial transcriptomics has localized FIAs near collagen-dense zones where TGF-β signaling induces fibrogenic reprogramming of cells [47]. FIAs produce excessive ECM components and PAI-1 in a manner that promotes vascular fibrosis and stiffening alongside VSMC proliferation [48]. Two additional human-specific adipocyte subtypes have been identified by single-cell omics. First, (4) senescent adipocytes expressing *CDKN2A* (p16INK4A), *CDKN1A* (p21CIP1), *SERPINE1* (PAI-1), and senescence-associated secretory phenotype (SASP) factors (e.g., IL-1α, IL-6, MMP3) have been identified in aged and diabetic PVAT, which are thought to promote local inflammation and tissue remodeling [49,50,51]. Next, (5) microvascular pericytes with *MYH11^−^/PDGFRB^+^/CD146^+^* genetic signatures exhibit dual contractile and adipogenic potential to modulate both angiogenesis and vascular tone [40,52]. Together, these findings from single-cell technologies offer a refined view of adipocyte diversity that is complemented by an equally complex immune and stromal landscape, all of which collectively orchestrate PVAT’s functional output.

### 2.2. Immune Ecosystem: Dynamic Regulators of Vascular Inflammation

Beyond adipocytes, the use of single-cell technologies has further revealed specialized immune niches in PVAT that dynamically respond to metabolic cues and play decisive roles in promoting vascular health and disease. Resident thermoregulatory macrophages (RTMs), characterized by *Lyve1^+^/MHC-IIˡᵒ/Tim4^+^* expression in healthy T-PVAT, support beiging through noradrenaline recycling [33,53,54]. Conversely, CD9^+^ lipid-associated macrophages (LAMs) form crown-like structures in obese A-PVAT, secreting factors such as interleukin-1β (IL-1β) and matrix metalloproteinase-9 (MMP9) that collectively degrade elastic laminae [36,55]. The T lymphocyte compartment of PVAT includes Foxp3^+^/IL-10^+^ regulatory T cells (Tregs) that suppress adipose inflammation, while pathogenic T helper 17 (Th17) cells (RORγt^+^/IL-17A^+^) expand in hypertension to promote vascular remodeling and fibrosis via interleukin-17A (IL-17A) signaling [56,57]. Cytotoxic CD8+ T cells in PVAT further contribute to atherosclerotic plaque instability through granzyme-mediated apoptosis [58,59]. Perivascular mast cells release chymase, which, in turn, activates angiotensin II to promote vasoconstriction, inflammation, and ECM degradation in diabetes [16,38]. Adding to this complexity, a spatially distinct population of septal LYVE1^+^ macrophages has been shown to control adipocyte stem cell (ASC) fate in white adipose tissue (WAT) via TGF-β1, thereby influencing adipose tissue plasticity and systemic metabolism [60]. Together, dynamic immune surveillance in PVAT is essential for tissue homeostasis, pathological tissue remodeling, and communication with neighboring vascular structures.

### 2.3. Stromal Architecture: Progenitors and Extracellular Matrix Regulators

The cellular component of PVAT is embedded within a structured stromal ECM scaffold that not only provides structural support but also actively regulates tissue mechanics and cellular fate. The stromal compartment of PVAT, comprising fibroblasts and progenitor cells, dictates the structural and signaling milieu that governs vascular mechanics and adipogenesis. Indeed, *Postn^+^/Tcf21^+^* fibromyocytes maintain mechanical homeostasis near the vascular adventitia through adaptive collagen production [61,62]. Consistent with this matrix-regulatory role, biomechanical testing coupled with single-nucleus RNA-seq reveals that collagen-integrin α5/β1 interactions mediate PVAT stress relaxation—a protective mechanism for dissipating cyclic vascular strain [4]. Dysregulation of this integrin-mediated mechanotransduction pathway in hypertension increases ECM stiffness and promotes VSMC dysfunction via YAP/TAZ mechanosensing [63].

In addition, perivascular *Dpp4^+^/Pref1^+^* adipogenic progenitors differentiate into adipocytes upon metabolic stress, thus serving as biological sensors of tissue damage [52,64]. Multi-omics integration demonstrates discordant regulation of *Scd1* in these cells: while *Scd1* mRNA increases in obese A-PVAT, protein levels decline due to ubiquitin-mediated degradation [41]. This gene-protein discordance disrupts lipid metabolism and amplifies NF-κB-driven inflammation in PVAT. Beyond adipogenic progenitors, PVAT contains neural-associated cell populations expressing markers such as *Sox10*, likely representing resident glial or Schwann cells [65]. Lineage-tracing studies have identified *Gli1^+^/Sca1^+^* adventitial progenitors capable of migrating within the vascular wall and adopting fibroblast or VSMC-like states during instances of vascular remodeling [66]. Single-nucleus multi-omics (snRNA-seq + snATAC-seq) has been used to delineate how the specific progenitors undergo pathological reprogramming in aneurysms [29]. To this end, epigenetic remodeling via EZH2-mediated H3K27me3 deposition silences contractile genes in VSMCs (e.g., *Myh11*, *Acta2*), while open chromatin at the *KLF4* loci promotes formation of synthetic VSMC phenotypes [29,67]. The functional specialization of these cellular compartments within PVAT is not uniform for all adipose depots but is critically defined by their anatomical location, leading to differing roles for adipogenic progenitors in T-PVAT and A-PVAT.

### 2.4. Region-Specific Cell Communication Drives Functional Diversity

The cellular building blocks of PVAT are organized into distinct functional units across anatomical depots, with T-PVAT specializing in thermoprotection and A-PVAT exhibiting a propensity for inflammation. Taken together, this leads to profound functional perivascular divergence along the aorta [1,2]. T-PVAT maintains thermogenic competence via TAP-derived beige adipogenesis, which is thought to limit cardiovascular disease burden [8,68]. Indeed, mitochondrial abundance in T-PVAT correlates inversely with atherosclerosis severity [23,43]. Specific anti-atherogenic mechanisms include UCP1-dependent fatty acid oxidation (reducing local free fatty acid (FFA) availability), secretion of vasodilatory nitric oxide and adiponectin, and catecholamine-induced atheroprotective gene expression by VSMCs [16,24]. Furthermore, the distinct secretome of browning adipocytes within T-PVAT provides critical structural protection to the adjacent vessel wall by reducing inflammation, directly inhibiting VSMC apoptosis, and preventing aortic aneurysm formation [69,70]. Single-cell spatial mapping reveals “immuno-thermogenic niches” where RTMs directly contact beige adipocytes to facilitate noradrenaline reuptake and maintain homeostatic levels of vascular tone [33,54].

In contrast, A-PVAT demonstrates a predisposition toward inflammatory reprogramming [2,6,12,70]. Metabolic dysregulation, primarily characterized by adipose tissue insulin resistance, triggers uninhibited adipocyte lipolysis, which releases FFAs that activate toll-like receptor 4 (TLR4)-mediated inflammatory signaling and drive macrophage recruitment and polarization toward CD9^+^ populations [6,35,36]. The resulting inflammatory milieu (e.g., IL-1β, TNF-α, MCP-1) within PVAT induces paracrine effects on EC activation, VSMC proliferation, and ECM degradation—key processes in aneurysm formation and the development of hypertension [48,55,71]. Increased ECM stiffness in hypertensive A-PVAT alters integrin β1 signaling to promote VSMC switching to a synthetic phenotype [4,63,72]. Spatial transcriptomics of human A-PVAT shows overlapping zones of *LEP*^+^ adipocytes and *IL1B*^+^ macrophages surrounding sclerotic vessels, suggesting an interplay between white adipocytes, inflammation, and vascular disease [38,73]. Under the pressure of chronic disease, these adipose depot-specific cellular landscapes undergo profound and coordinated pathological transformations that further exacerbate vascular dysfunction.

### 2.5. Pathological Transformation Reshapes Cellular Landscapes

Disease states catalyze a fundamental dysfunction and reorganization of the PVAT cellular landscape that reshapes cellular proportions, activation states, and communication networks to drive the initiation and progression of pathology [6,18,48]. During obesity progression, for example, macrophage populations in PVAT undergo dramatic polarization from protective M2 phenotypes to inflammatory M1 states, while T cell infiltration into PVAT increases with enrichment of pro-inflammatory Th1 and Th17 populations [20,36,71]. These immune changes coincide with fibroblast activation and enhanced collagen production, creating a profibrotic microenvironment that predisposes to compromised vascular function [21,48,74,75]. Fibroblast activation enhances collagen production, while adipocyte transdifferentiation generates fibro-inflammatory phenotypes in dysfunctional PVAT [46,70,72]. This activation also precedes further immune recruitment, with ECM stiffening triggering macrophage TLR4 signaling via integrin clustering [35,48,72]. Temporal analyses reveal that early immune infiltration initiates self-sustaining pathogenic loops that ultimately drive irreversible ECM remodeling in the PVAT-vascular complex [1,18,35,76].

Cardiovascular pathology triggers disease-specific cellular adaptations in PVAT. In atherosclerosis, perivascular *SPP1*^+^ macrophage accumulation precedes atherosclerotic plaque formation and correlates with stenosis severity [27,30]. Hypertension induces sex-specific immune changes, with increased CD4^+^ memory T cells in males and enhanced M2-like macrophages in females, highlighting the importance of considering biological sex in PVAT and cardiovascular research [56,77,78,79,80]. Complementing these experimental observations, recent histopathological evidence demonstrates that age and biological sex significantly modify the association between dyslipidemic profiles and carotid atherosclerotic plaque instability in humans. While this study did not directly assess PVAT, these clinical findings further underscore the critical need to incorporate both sex and age as biological variables in future human PVAT studies focused on evaluating the relationship between metabolic risk factors and vascular remodeling [81]. Collectively, these findings illustrate that pathological transformation of PVAT is not a passive consequence but an active and coordinated recalibration of the PVAT cellular ecosystem that potentiates a self-amplifying cycle of inflammation and fibrosis to drive progressive vasculopathy.

In summary, multimodal single-cell atlases have redefined PVAT as a complex, multi-cellular ecosystem by revealing specialized adipocyte, immune, and stromal subpopulations and their biological functions in health and disease. The cellular diversity of PVAT is organized into distinct functional units across anatomical depots along the aorta, with thermogenic T-PVAT contrasting sharply with inflammation-prone A-PVAT. In disease, this system undergoes a pathological transformation where coordinated shifts in cellular composition and communication create a self-amplifying loop of inflammation and fibrosis. This refined cellular atlas provides the essential foundation for mechanistically decoding PVAT-vascular crosstalk and identifying novel therapeutic targets.

## 3. Cell Plasticity and Phenotypic Transitions

**Figure 2:**
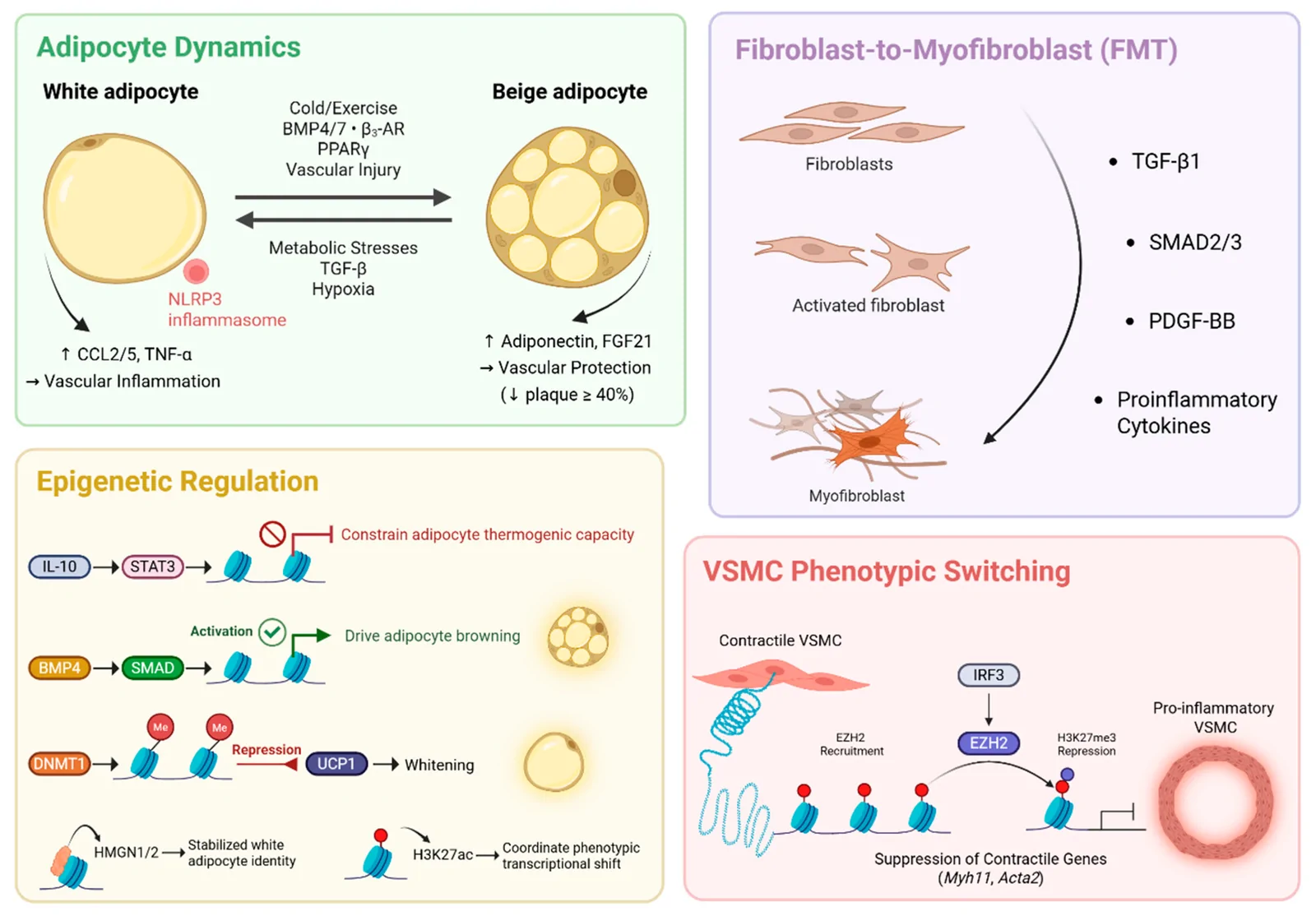
Molecular and epigenetic circuits drive cellular plasticity and phenotypic transitions at the PVAT-vascular interface. The schematic illustrates the dynamic, stimulus-driven reprogramming of major adipocyte, stromal, and vascular lineages at the PVAT-vascular interface. Adipocyte Dynamics: The bidirectional plasticity between lipid-storing white adipocytes and thermogenic beige adipocytes represents a critical metabolic switch. Fibroblast-to-Myofibroblast Transition (FMT): Stromal fibroblasts undergo profibrotic activation into myofibroblasts, a process driven by TGF-β, SMAD2/3, PDGF-BB, and immune-derived proinflammatory cytokines, that contribute to ECM deposition and tissue fibrosis. Epigenetic Regulation: Chromatin-level modifications dictate the cellular identity of adipocytes in PVAT. IL-10/STAT3 signaling constrains thermogenic capacity, while the BMP4/SMAD axis activates browning programs in adipocytes. In contrast, DNMT1-mediated methylation represses UCP1 to drive adipocyte whitening. Nucleosome-binding proteins (HMGN1/2) stabilize white adipocyte identity, while dynamic enhancer acetylation (H3K27ac) coordinates broader phenotypic transcriptional shifts. VSMC Phenotypic Switching: Within the vascular wall, inflammatory signaling (e.g., via IRF3) recruits the epigenetic modifier EZH2 to deposit repressive H3K27me3 marks at the promoters of contractile genes (*Myh11*, *Acta2*) in VSMC, driving the transition from a healthy contractile state to a pro-inflammatory, synthetic phenotype.

PVAT cellular plasticity, governed by metabolic, mechanical, and inflammatory cues, underpins its shift from vasoprotective to pathogenic states [44,82]. Multimodal single-cell technologies now have the capability to resolve these transitions at unprecedented resolution, revealing the molecular circuits controlling adipocyte browning/whitening, fibroblast-myofibroblast differentiation, immune reprogramming, and VSMC phenotypic switching [38,58] (Figure 2).

### 3.1. Adipocyte Dynamics: Browning Versus Whitening

The balance between adipocyte browning and whitening represents a critical axis in PVAT’s functional identity that directly influences vascular health. Browning (or beiging) can be induced by cold exposure, exercise, BMP4/7, β3-adrenergic agonists (e.g., mirabegron), or PPARγ ligands [9,19,43,83,84]. Mechanistically, this requires activation of the *Ppargc1α*-*Ucp1* signaling axis, mitochondrial biogenesis, and suppression of Notch signaling. Single-Nuclei Adipocyte RNA Sequencing (SNAP-seq) has overcome technical barriers in profiling lipid-rich adipocytes and has successfully identified 14 transcriptionally distinct adipocyte populations in PVAT [85]. Among these, there was emergence of a distinct subpopulation of highly active thermogenic adipocytes characterized by robust expression of β3-adrenergic receptors, hormone-sensitive lipase, and thermogenic genes including *Ucp1*, *Cidea*, and *Dio2*. Cold exposure and β3-adrenergic stimulation dramatically increased the abundance of this adipocyte subpopulation from 3.4% to 23.5% in PVAT, demonstrating remarkable dynamic cellular plasticity in response to metabolic demands [85]. Spatial transcriptomics further revealed that BMP4-mediated browning requires SMAD1/5/8 recruitment to induce *Ppargc1a* transcription and enhance fatty acid oxidation (FAO) [43,86]. The process of adipose beiging converts lipid-storing adipocytes to mitochondria-rich thermogenic adipocytes capable of secreting beneficial adipokines (e.g., adiponectin, FGF21) that can improve vascular reactivity and substantially reduce plaque burden in murine models [19,43,84]. A recent discovery of vascular injury-induced PVAT beiging represents a paradigm-shifting finding that reframes our understanding of adipose-vascular crosstalk. Single-cell analysis revealed that vascular injury triggers acute PVAT inflammation followed by a phase of adipose beiging that acts as a protective homeostatic response [87]. In particular, F4/80+ macrophages infiltrate PVAT within 24 h of injury and induce brown adipose tissue (BAT)-like phenotypic changes in PVAT through mechanisms involving PRDM16-dependent regulation [19].

In contrast, adipose whitening can be triggered by metabolic stress (e.g., hyperglycemia, hyperlipidemia), TGF-β superfamily ligands (e.g., BMP3, activin A), or hypoxia [2,6,12,18,50]. This process suppresses UCP1 and oxidative phosphorylation while enhancing adipocyte lipogenesis and cytokine production [6,18]. Spatial lipidomics revealed that ceramide accumulation in whitened adipocytes activates NLRP3 inflammasomes [36,49]. Thus, the adipose whitening process creates a self-amplifying pathological loop of adipocyte inflammation where (1) lipid-laden adipocytes secrete chemokines (e.g., CCL2, CCL5) to recruit monocytes [88], (2) macrophage-derived TNF-α further suppresses *Ucp1* via NF-κB signaling [89], and (3) adipocyte-derived fatty acids activate macrophage NLRP3 inflammasomes to promote adipocyte inflammation [36,49]. Consequently, whitened PVAT becomes a reservoir of inflammation capable of accelerating adjacent vascular pathology [2,20,23]. Therefore, the adipose browning/whitening axis represents a reversible switch whose position dictates whether PVAT serves as a metabolic sink or an inflammatory reservoir and provides a level of cellular plasticity that extends to the stromal compartment where fibroblasts similarly undergo fate-altering transitions.

### 3.2. Fibroblast-to-Myofibroblast Transition (FMT)

PVAT-resident fibroblasts exhibit extraordinary phenotypic plasticity that underlies tissue remodeling in health and disease [15,61,87]. Single-cell transcriptomics (scRNA-seq) trajectory analysis of PVAT identified differentiation of *Dpp4*^+^ progenitors into αSMA^+^ myofibroblasts during hypertension [40,47]. TGF-β1 induces this shift through SMAD2/3-dependent *Acta2* activation [67,90]. Similarly, immune-derived cytokines can trigger fibroblast-to-myofibroblast (FMT) transition states that foster fibrosis and excess ECM remodeling in the context of abdominal aortic aneurysms [91].

Multi-omics integration has identified FSP1^+^ fibroblasts as critical cells in the adipogenic niche that create structural microenvironments for adipocyte precursors [25,92,93]. Despite lacking intrinsic adipogenic potential (PPARγ^−^/CEBPα^−^), these specialized fibroblasts are essential for maintaining preadipocyte pools in PVAT through PDGF-BB-mediated stromal-epithelial crosstalk [37,52,92,93,94]. scRNA-seq confirms FSP1^+^ fibroblasts express high levels of matrix-organizing genes while also secreting PDGF-BB to support adipogenic differentiation [93]. Cardiovascular disease states trigger disease-specific fibroblast phenotypic transitions. For example, scRNA-seq and spatial transcriptomics have demonstrated that spatially resolved macrophage-fibroblast interactions promote myofibroblast differentiation, contributing to tissue fibrosis and vascular dysfunction [95,96,97]. These findings highlight the importance of immune-stromal cell interactions in driving pathological tissue remodeling.

### 3.3. Epigenetic Regulation of Cellular Identity

Single-cell epigenetic analysis has revealed the chromatin-level mechanisms controlling PVAT cellular plasticity. In particular, IL-10 signaling establishes an immune-metabolic axis that directly constrains adipocyte thermogenic capacity by limiting chromatin accessibility at key sites that promote beiging genes (e.g., *Ucp1*, *Ppargc1*, *Cidea*, *Dio2*) [85]. Additionally, the nucleosome-binding proteins HMGN1/2 stabilize white adipocyte identity by maintaining chromatin architecture, while H3K27ac dynamics at specific enhancers coordinate the transcriptional shifts during adipose phenotypic transitions [98]. Mechanistic investigation into the regulation of adipose phenotypic plasticity in PVAT has uncovered metabolic-epigenetic circuits driving adipocyte browning through BMP4-SMAD/UCP1 activation [43,86] and whitening via DNMT1-mediated *Ucp1* repression [25,99].

Transcriptional regulators including *Prdm16*, *Nfia*, and *Ebf2* act as master genetic controllers of adipose beiging through their ability to regulate chromatin modifications that facilitate environmental responsiveness [8,100,101,102,103]. These factors co-localize with transcriptional machinery to modulate cell-specific gene expression programs, where changes in chromatin accessibility occur in response to thermogenic stimuli to enable rapid transcriptional responses [103]. This layer of epigenetic regulation is equally critical in the adjacent vascular wall itself, where it governs the fate of VSMCs, a key determinant of vascular stability and disease progression.

### 3.4. VSMC Phenotypic Switching: Epigenetic-Metabolic Control

Since PVAT and the aortic wall function as a bidirectional signaling unit, VSMC state transitions may also contribute to signaling in the adjacent PVAT niche. To this end, single-nucleus multi-omics (snRNA-seq + snATAC-seq) of human aneurysmal tissues have delineated epigenetic drivers associated with disease presentation. In particular, IL-1β activates STING-IRF3 signaling, which recruits EZH2 to deposit H3K27me3 at contractile gene (e.g., *Myh11*, *Acta2*) loci, thereby regulating VSMC phenotypic shifts [67,104]. scRNA-seq further revealed that deletion of the long, ubiquitously expressed isoform of with-no-lysine kinase 1 (L-WNK1) leads to a proinflammatory VSMC phenotype, increased perivascular immune cell infiltration, and significant aortic dilation with impaired contractility [57]. Studies in murine models demonstrate that PVAT macrophage polarization shifts during vascular injury [19]. Early phases are dominated by acute inflammatory responses and subsequent attempts at resolution by reparative MHC-II^hi^ macrophage subsets; pathogenic CD9^+^ subsets that are highly conserved in human adipose tissue ultimately emerge to amplify late-stage damage in response to vascular injury [33,34,53].

Single-cell technologies have provided cell-resolved insight into plasticity of the PVAT ecosystem by defining transcriptional states and regulatory programs associated with cellular activation and transition. These transitions include adipose beiging/whitening, fibroblast activation, and VSMC phenotypic switching, which are all governed by interconnected metabolic, inflammatory, and epigenetic circuits. However, these cellular fate decisions do not occur in isolation. They are coordinated through dense intercellular communication networks that spatially organize the PVAT-vascular interface. Having established the cellular composition in PVAT and its plasticity in response to stressors, we now dissect how their communication networks become dysregulated to drive disease, moving from cell-intrinsic mechanisms to intercellular pathophysiology.

## 4. Adipocyte-Vascular Crosstalk in Disease: Intercellular Communication Networks

**Figure 3:**
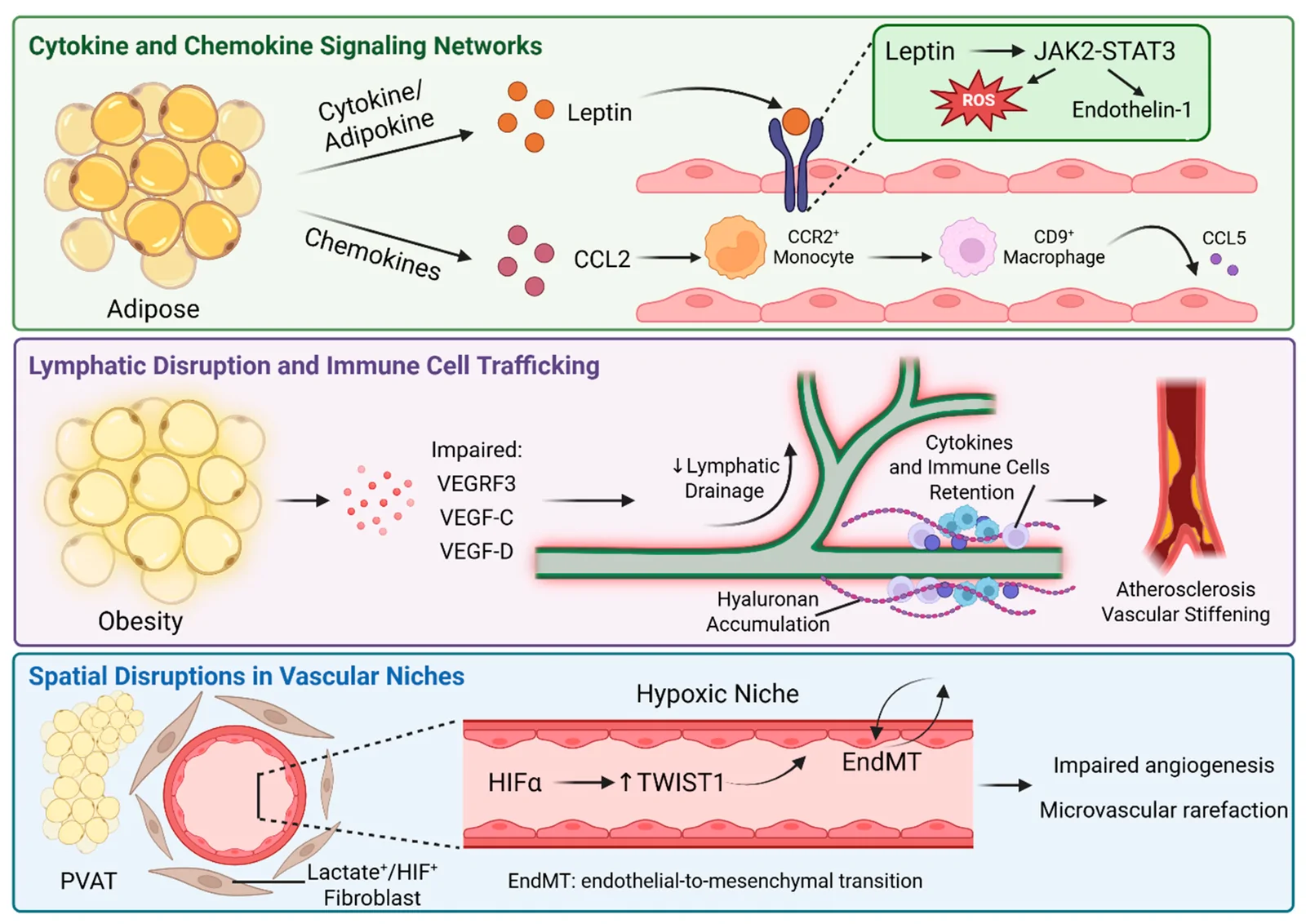
Pathological intercellular communication networks and spatial disruptions at the PVAT-vascular interface. Single-cell and spatial technologies map the dysregulated signaling circuits driving cardiometabolic disease. (**Top**) Cytokine and Chemokine Signaling Networks: Dysregulated adipocytes initiate paracrine and immune crosstalk. Adipocyte-derived leptin activates endothelial JAK2-STAT3 signaling, increasing reactive oxygen species (ROS) and endothelin-1 production to induce endothelial dysfunction. Adipocyte-derived CCL2 recruits circulating CCR2^+^ monocytes, which differentiate into inflammatory CD9^+^ macrophages and subsequently secrete CCL5 to create a self-amplifying loop of localized immune recruitment. (**Middle**) Lymphatic Disruption and Immune Cell Trafficking: Obesity physically and molecularly impairs the perivascular lymphatic drainage system. This mechanical failure causes retention of pro-inflammatory cytokines and immune cells within the perivascular space that can directly accelerate atherosclerosis and vascular stiffening. (**Bottom**) Spatial Disruptions in Vascular Niches: Spatial multi-omics identifies localized clusters of lactate^+^/HIF1α^+^ fibroblasts adjacent to capillaries. Within these hypoxic microenvironments, HIF1α stabilization upregulates TWIST1, actively driving the endothelial-to-mesenchymal transition (EndMT). This spatially organized pathological remodeling results in microvascular rarefaction and impaired angiogenesis, leading to further exacerbated tissue hypoxia and irreversible vascular dysfunction.

The cellular plasticity and diversity within PVAT is functionally integrated through a complex web of intercellular communication. Single-cell and spatial technologies have moved beyond cataloging cell types and are now able to map the precise cytokine networks, extracellular vesicle trafficking, and spatially organized niches that mediate PVAT dysfunction in cardiometabolic disease states [35,51,105]. By integrating transcriptomic, spatial, and functional data, we now resolve how these defined molecular pathways within PVAT drive vascular pathology (Figure 3).

### 4.1. Cytokine and Chemokine Signaling Networks

Single-cell secretome analysis using CellChat and NicheNet has successfully mapped disease-specific signaling pathways to reveal how dysregulated PVAT-vascular signaling initiates and perpetuates disease [106,107]. In diabetic PVAT, adipocyte-derived leptin activates endothelial JAK2-STAT3 signaling, leading to the production of reactive oxygen species and endothelin-1 that promote vasoconstriction and disruption of barrier function [9,40,72,108]. Spatial transcriptomics demonstrates compartment-specific signaling via confirmation of leptin receptor (*LEPR*) enrichment in ECs present in the lumen of sclerotic vessels [73,109,110]. Complementing this observation, C-reactive protein (CRP) produced by PVAT enhances adventitial macrophage infiltration and vasa vasorum proliferation to exacerbate neointima formation after vascular injury [111]. Within αSMA-positive VSMCs, PVAT-derived CRP also upregulates MMP2/9 activity to accelerate aneurysm progression [112]. Furthermore, a critical scRNA-seq discovery involves the IL10 regulatory axis in adipocytes, wherein lymphocyte-derived IL-10 acts directly on the adipocyte IL10Rα to antagonize thermogenesis and maintain energy homeostasis [85]. Under β3-adrenergic stimulation to induce browning, B-cells show a 3-fold increase in IL-10 expression, likely creating a negative feedback loop that prevents excessive lipolysis during transient instances of high energy demand [85].

Similarly, neuregulin 4 (NRG4) is a batokine secreted specifically by beige adipocytes that functions as a key anti-inflammatory mediator in PVAT [19,113]. Single-cell atlases confirm NRG4 signals through ErbB4 receptors to drive alternative macrophage activation (M2 polarization) [24,114] and suppress TNF-α, IL-6, and CCL2 production while simultaneously enhancing expression of *Il10* and *Arg1* [24,115]. This specific beige PVAT axis facilitates the resolution of vascular inflammation and reduces EC activation [113,114]. Chemokine gradients across PVAT depots coordinate leukocyte trafficking, as demonstrated by time-resolved scRNA-seq showing the ability of adipocyte-derived CCL2 to recruit CCR2^+^ monocytes capable of differentiating into CD9^+^ macrophages in obese PVAT [34,36]. These macrophages subsequently secrete CCL5 to amplify leukocyte and lymphocyte recruitment [71]. Spatial proteomics further demonstrated that macrophage migration inhibitory factor (MIF) secreted by inflamed PVAT binds endothelial CD74 and contributes to exaggerated lipid accumulation and atherosclerosis progression [30]. Beyond soluble factors, the trafficking and positioning of immune cells themselves are regulated by a specialized lymphatic system in PVAT, whose dysfunction in obesity and other pathologic states creates a permissive environment for chronic inflammation.

### 4.2. Lymphatic Disruption and Immune Cell Trafficking

The bidirectional crosstalk between lymphatic endothelial cells (LECs) and adipose tissue provides additional links between PVAT dysfunction and cardiovascular pathology [84]. Single-cell analysis reveals LECs maintain adipose tissue metabolic homeostasis through lipid transport and immune surveillance [116,117]. In particular, obesity-induced VEGFR3 downregulation impairs lymphatic function in PVAT and exacerbates inflammation and vascular stiffening [118,119]. Integrated single-cell lymphatic atlases reveal that reduced VEGF-C in obese PVAT drives cytokine accumulation by impairing lymphatic drainage [1,54,84]. This was further confirmed using *Prox1*-tdTomato reporter mice, where lymphatic dysfunction accelerated atherosclerosis via excess immune cell retention in PVAT [120,121].

Immune trafficking and clearance via the embedded lymphatic system maintains tissue homeostasis; instances of dysfunction drive chronic inflammation through immune cell retention in tissues and adipose depots [122]. Adipocyte-derived VEGF-D promotes lymphangiogenesis, but its effects are context-dependent; it improves tissue drainage and reduces inflammation in lean states, but hyaluronan accumulation blunts VEGF-D responsiveness and exacerbates PVAT immune cell accumulation in obesity [84,117]. Consequently, therapeutic strategies aimed at promoting lymphangiogenesis to enhance immune clearance and lower inflammation may need to be tailored to the underlying metabolic environment and likely require the prior resolution of fibrotic barriers, such as the excess hyaluronan seen in obese patients.

Alongside these structural drainage networks, multi-omics techniques have highlighted how resident immune cells actively dictate the inflammatory milieu. The discovery of Type 2 innate lymphoid cells (ILC2s) as resident cells in visceral adipose tissue has revealed their crucial role in maintaining eosinophil and alternatively activated macrophage populations [123]. ILC2-derived IL-5 and IL-13 production promote eosinophil accumulation and M2 polarization, which effectively suppresses inflammation and enhances insulin sensitivity [123,124]. This axis is amplified during parasitic helminth infections, where ILC2 expansion improves glucose tolerance [125]. The functional output of these signaling and trafficking networks is ultimately determined by their spatial configuration within the tissue, noting that cardiometabolic disease states reshape the anatomical niches of PVAT-vascular interaction.

### 4.3. Spatial Disruptions in Vascular Niches

Multimodal spatial omics approaches have begun to map disease-induced reorganization in PVAT. Indeed, spatial transcriptomics has revealed lactate^+^/HIF1α^+^ fibroblast clusters adjacent to capillaries in hypertensive PVAT that correlate with impaired angiogenesis [29,50,72,73]. CODEX-like high-throughput immunoprofiling of human atherosclerotic plaques identified B-cell follicles with expanded B-cell receptor repertoires near VEGFC^+^ fibroblasts that collectively form tertiary lymphoid organ structures [27,58]. Three-dimensional MERFISH quantification demonstrated increased macrophage-adipocyte contacts in diabetic PVAT that enhance IL-1β transfer [35,36,73]. These spatially resolved mechanisms, uncovered exclusively through spatial omics techniques, reveal the formation of pathological feedback loops. For instance, HIF1α stabilization in stiffened ECM zones induces endothelial-to-mesenchymal (EndMT) transition through TWIST1 upregulation while suppressing VEGFA-dependent reparative angiogenesis [50,110,126]. The resulting microvascular rarefaction in PVAT exacerbates tissue hypoxia to establish self-amplifying cycles of local vascular dysfunction [127,128].

The convergence of single-cell spatial multi-omics and functional validation experiments has transformed our understanding of PVAT-vascular crosstalk from one of descriptive association to one of mechanistic causality. By mapping signaling circuits at cellular resolution, we can now identify targetable nodes, such as the CCR2-CCL5 axis in monocyte recruitment [34,52] or MIF-CD74 in EC activation [27,29], that drive disease progression across metabolic and cardiovascular conditions [40,129]. These advances provide the foundation for spatially precise interventions that disrupt pathological communication networks in PVAT while preserving physiological crosstalk between neighboring tissues.

## 5. From Single-Cell Insights to Clinical Strategies: A Roadmap

By resolving PVAT’s cellular heterogeneity and mechanisms of dysfunction, single-cell profiling has pinpointed actionable biomarkers and potential therapeutic targets for intervention. Translating these discoveries, however, requires navigating a path from mechanistic insight to clinical application. This section synthesizes the immediate therapeutic opportunities emerging from single-cell PVAT atlases, critically evaluates the persistent translational challenges, and outlines a forward-looking technological roadmap to establish PVAT-targeted precision medicine.

### 5.1. Emerging Biomarkers and Mechanism-Based Therapies

Single-cell atlases have moved beyond characterization of biological mechanisms to the identification of actionable biomarkers for early detection and strategies for therapeutic intervention. Leveraging single-cell transcriptomics, preadipocyte clusters have been identified as sentinels of vascular dysfunction. scRNA-seq of human pre-diabetic PVAT revealed DLK1 downregulation in *DPP4*^+^/*PI16*^+^ preadipocytes, which was consistent with future vascular impairment [52,130]. Complementing this, *SPP1*^+^ macrophages represent another high-value therapeutic target identified through single-cell analysis of PVAT in the context of atherosclerosis. These osteopontin-expressing cells promote fibrosis and extracellular matrix remodeling [27,131]; however, therapeutic targeting via CXCL4 inhibition reduces *SPP1*^+^ macrophage differentiation and ameliorates fibrosis [30].

Using analysis of liquid biopsies from whole blood collection, circulating extracellular vesicles (EVs) from diabetic PVAT carrying the microRNA miR-34a correlate with inflammation and predict incidence of cardiovascular events [132,133]. Obese adipocyte-derived miR-34a is thus a potential therapeutic target that shuttles bioactive molecules into recipient macrophages to promote pro-inflammatory M1 polarization and inhibit anti-inflammatory M2 polarization, which directly links to cardiovascular complications in diabetic conditions [132,134]. Another compelling microRNA target, miR-378a-5p, was revealed by single-cell RNA sequencing of PVAT-derived mesenchymal stem cells and was found to contribute to metabolic reprogramming of VSMCs and vascular regeneration through CDK1/p21 pathway regulation [135].

These biomarkers are complemented by mechanism-based therapies such as metabolic reprogramming agents that leverage single-cell insights into adipocyte plasticity. Specifically, recombinant BMP4 induces adipocyte browning through SMAD1/5-dependent *Ucp1* activation, and treatment has been shown to reduce atherosclerotic plaque burden in preclinical animal models via enhanced lipid clearance and M2 macrophage polarization [43,86]. Similarly, pioglitazone activates PPARγ in obese mice to reduce aortic PVAT inflammation and improve EC function without directly altering PVAT mass or phenotype (e.g., browning) [9].

Immunomodulatory strategies are another approach to target pathological immune circuits previously revealed by single-cell mapping. Finerenone, a non-steroidal mineralocorticoid receptor antagonist, reduces expression of pro-contractile prostaglandins, reactive oxygen species (ROS), and inflammatory cytokines in PVAT and perirenal adipose tissue, which collectively attenuates perivascular fibrosis in diabetic vasculature [136]. The cardiovascular and renal protective effects of finerenone have been demonstrated in recent phase III clinical trials (e.g., FIDELIO-DKD) and highlight the translational viability of dampening these localized perivascular inflammatory pathways [137,138,139]. Similarly, propagermanium blocks monocyte recruitment to PVAT via CCR2 inhibition, which works to restore EC function in type 2 diabetes by disrupting the CCL2-CCR2 signaling axis identified through time-resolved scRNA-seq [140]. Anti-IL-1β therapy mitigates PVAT inflammation, and the associated NLRP3 inhibition reduces diabetic vascular aging [49]. Clinically, anti-IL-1β therapy with canakinumab reduced recurrent cardiovascular events in the CANTOS trial partly through dampening lipid inflammation in patients with coronary artery disease [141]. Taken together, development of a safe and effective approach co-targeting NLRP3 (finerenone) and CCR2 (propagermanium) may amplify benefits in diabesity-associated vasculopathy.

Collectively, these advances establish a diverse and promising therapeutic pipeline that spans sensitive cellular sentinels and liquid biomarkers to targeted metabolic, immunomodulatory, and epigenetic agents.

### 5.2. Current Translational Challenges

Despite a promising pipeline for therapeutic intervention in PVAT, the translation of single-cell findings into clinical practice is impeded by significant biological, technical, and clinical hurdles.

#### 5.2.1. Species-Specific Biology and Human Relevance

A primary biological barrier impeding clinical translation is the existence of species-specific differences in adipocyte biology. Murine PVAT exhibits substantially greater browning capacity than human tissue, with comparative single-cell transcriptomics revealing higher *Ucp1* expression in mice than in humans [32,39]. This fundamental divergence limits extrapolation of browning agent efficacy from preclinical models to human applications. To address this translational gap, future development of humanized mouse models with engrafted human PVAT may bridge the differences in browning efficacy by providing more physiologically relevant platforms for therapeutic validation. Functional human adipose tissue has been successfully maintained after xenotransplantation into immunodeficient mice, suggesting that adapting this approach to human PVAT could provide a more physiologically relevant platform for therapeutic evaluation [142,143]. Species-specific differences in adipocyte-derived EV biomarker expression have also been reported, underscoring the need to validate the identification of murine EV biomarkers in human PVAT and plasma [144]. Emerging studies demonstrate that circulating adipose-derived EV profiles can correlate with tissue-level inflammatory parameters. Liquid biopsy approaches have shown promise for isolating tissue-specific EVs from blood based on unique protein signatures [145] potentially enabling non-invasive assessment of vascular inflammation in the future.

#### 5.2.2. Therapeutic Specificity and Side Effects

Therapeutic side effects require mitigation strategies when considering delivery in humans. For example, PPARγ agonists improve PVAT biology by reducing inflammation; however, pioglitazone causes fluid retention and weight gain in clinical applications [9] and finerenone therapy carries a hyperkalemia risk [136]. These limitations are driving accelerated development of nanoparticle-based drug delivery systems designed to target specific perivascular cell types (e.g., macrophages) in an effort to substantially enhance delivery precision and reduce off-target effects compared to broad systemic administration [146].

#### 5.2.3. Clinical Implementation and Standardization

Finally, clinical implementation frameworks must address cost-effectiveness and standardization barriers. For example, the proposed Fat Attenuation Index (FAI) biomarker shows improvement in patient survival risk; however, its value proposition requires balancing imaging costs against reduced unnecessary treatments [21,23,147]. Namely, determination of the FAI requires routine coronary computed tomography angiography (CCTA) to quantify spatial changes in PVAT radiodensity, measuring shifts in Hounsfield units that reflect localized inflammation and edema [148,149]. Following the precedent set by FDA clearance of the FAI for cardiovascular risk assessment, regulatory pathways for clinical implementation should prioritize comprehensive biomarker qualification through four critical phases: (1) analytical validation, (2) establishment of biomarker sensitivity/specificity, (3) clinical validation confirming disease-stratification capability, and (4) demonstration of biomarker utility in a patient population. Addressing these multifaceted challenges by spanning biological relevance, therapeutic safety, dynamic monitoring, and clinical integration is required for harnessing the full clinical and therapeutic potential of PVAT biology.

### 5.3. Future Frontiers: Integrated Technologies for Precision Targeting

To move basic PVAT research toward clinical impact, we posit three interconnected frontiers intended to transform vascular adipose biology from descriptive science on a benchtop to targetable pathophysiology in the clinic.

#### 5.3.1. High-Resolution Spatial and Dynamic Mapping

To decode spatial contexts within PVAT, integrated spatial platforms can be leveraged to resolve zones of specific signaling microenvironments by combining CODEX multiplexed protein imaging, Xenium subcellular RNA mapping, and mass spectrometry-based in situ metabolomics to simultaneously profile >100 analytes across PVAT-vascular interfaces [27,28,29,40,73,95,150]. This approach identifies pathological microdomains, such as leptin-enriched adipocyte clusters near inflamed endothelia, and can be readily enhanced through AI-driven spatial pattern recognition, classification, and processing [48,95,150]. ECM multi-omics further integrates advanced biomechanical profiling with spatial transcriptomics to define biological processes sensitive to mechanical stimulus, such as the ability of collagen crosslinking and proteoglycan composition to regulate PVAT mechanosensing and reveal, for example, how fibrosis alters integrin β1 signaling in hypertension [4,47,63,151].

Coupled with single-nucleus multimodal sequencing (snRNA-seq + snATAC-seq), these tools will be able to delineate chromatin landscape dynamics during adipocyte browning/whitening and identify elements, such as the *UCP1*-associated enhancer, as therapeutic targets for metabolic reprogramming [29,99,100,152]. CRISPR-based lineage tracing has been shown to track PVAT progenitor fates across disease progression and ultimately resolve how *Pdgfra*^+^ progenitors, among others, differentially contribute to either perivascular fibrosis or thermogenesis in response to hemodynamic stress [8,52,74].

Furthermore, advanced intravital imaging, including high-speed multiphoton microscopy, can capture real-time dynamics of leukocyte adhesion and EV trafficking at PVAT-endothelial interfaces in awake animals. Such approaches have revealed transient interactions within tissues, such as calcium wave dynamics, that are otherwise undetectable in static analyses [40,51,69]. Similarly, in situ photoacoustic imaging with targeted contrast agents has enabled noninvasive visualization of PVAT oxygenation, lipid content, and macrophage burden, which could provide a potential image-based approach for longitudinal monitoring of PVAT-specific therapeutic responses in murine models and human trials [23,72].

#### 5.3.2. Human-Relevant Models for Therapeutic Validation

To bridge the human relevance gap associated with animal studies, a concerted effort is needed in human biomodeling. Multi-center human PVAT biobanking initiatives could be implemented to standardize collection protocols across thoracic/abdominal aortic PVAT depots with paired blood samples, clinical metadata, and multi-omics profiling. This sample collection scheme has been pioneered by international consortia such as PVAT-NET [25,38,44]. At a smaller scale, microphysiological systems incorporating human PVAT-vascular co-cultures under hemodynamic flow can recapitulate pathological crosstalk, with artery-on-chip models previously demonstrating EndMT transition in response to stiffened PVAT matrices and its association with dysregulated integrin signaling [4,47]. Complementing this microfluidic approach, the future development of patient-derived iPSC models that differentiate into PVAT-specific adipocytes, while accommodating CRISPR-engineered mutations (e.g., *APOE4*, *PCSK9*), will enable high-throughput screening of genotype-specific PVAT therapies. Such advanced platforms could be directly deployed to target recently identified pathogenic nodes in PVAT, including NLRP3 inflammasome activation or mitochondrial dysfunction in cardiometabolic diseases [49,50].

#### 5.3.3. AI-Driven Mechanism Study and Clinical Translation

Looking forward, AI offers potential conceptual frameworks to navigate biological complexity, particularly when grounded in high-fidelity models of cellular behavior, such as with so-called “virtual cells” [153]. These computational models predict a cell’s functional response to perturbation, explain it via molecular mechanisms, and discover novel biology through in silico hypothesis generation [153]. By training AI and machine learning (ML) models on massive interventional datasets, such as those from paired high-throughput transcriptomics, proteomics, and functional screening studies performed in PVAT, researchers can create these virtual constructs [154]. Establishment of such a virtual platform capable of simulating the PVAT ecosystem could theoretically enable the in silico modeling of complex cellular and molecular perturbations [155], such as combinatorial drug treatments to map pathogenic signaling circuits and identify key therapeutic nodes, with unprecedented speed.

Building on this idea of in silico model development, recent advancements in network pharmacology employ graph neural networks to integrate single-cell ligand-receptor pairs and predict synergistic drug combinations, such as BMP4 alongside CCR2 inhibitors, that concurrently enhance thermogenesis and suppress monocyte recruitment [43,106,107,154]. Additionally, clinical trial enrichment strategies can leverage AI-derived biomarkers from PVAT imaging (e.g., FAI radiomics) to identify responders within the patient population, as in the case of finerenone’s efficacy in diabetic cohorts with elevated PVAT inflammation [23,136,139,147]. Mechanobiological AI platforms can further predict ECM stiffness sensors from scRNA-seq atlases and identify therapeutic targets, such as integrin α5, for nanoparticle-mediated delivery of antifibrotic agents to pathological perivascular niches [4,63,146]. By integrating the predict-explain-discover capabilities of virtual cells with network pharmacology and clinical AI, the field can begin to transition from correlative biomarker discovery to a mechanistic, first-principles approach for target identification, treatment, and patient stratification. Ultimately, the goal of this proposed pipeline is to optimize clinical trials and accelerate the development of precision interventions for vascular-metabolic disease.

Collectively, these integrated strategies have the potential to catalyze the translation of single-cell discoveries into precision therapies that selectively disrupt pathological PVAT-vascular crosstalk while preserving metabolic homeostasis. Their eventual convergence could serve as the foundation for future clinical intelligence platforms that unify deep phenotyping, predictive AI, and targeted interventions to optimize and transform the management of vascular metabolic disease.

## 6. Conclusions: PVAT as a Therapeutic Nexus in Cardiovascular Precision Medicine

Multimodal single-cell technologies have redefined the established view of PVAT as a dynamic regulator of vascular health and disease by resolving its cellular heterogeneity, disease-associated states, regulatory programs, and spatial organization. These approaches have generated a comprehensive cellular atlas that has revealed PVAT depot-specific ecosystems, phenotypic plasticity, and pathological signaling circuits driving vasculopathy. To realize the clinical potential of these findings, future efforts should prioritize the integration of spatial multi-omics, lineage tracing, and computational modeling across diverse human cohorts. This concerted effort will help transition the field from descriptive cellular mapping to targeted therapeutics and will ultimately enable precise modulation of perivascular inflammation to improve cardiovascular outcomes.

## Data Availability

No new data were created or analyzed in this study. Data sharing is not applicable to this article.

## References

[1] Brown N.K., Zhou Z., Zhang J., Zeng R., Wu J., Eitzman D.T., Chen Y.E., Chang L. (2014). Perivascular Adipose Tissue in Vascular Function and Disease: A Review of Current Research and Animal Models. Arterioscler. Thromb. Vasc. Biol..

[2] Qi X.-Y., Qu S.-L., Xiong W.-H., Rom O., Chang L., Jiang Z.-S. (2018). Perivascular Adipose Tissue (PVAT) in Atherosclerosis: A Double-Edged Sword. Cardiovasc. Diabetol..

[3] Szasz T., Webb R.C. (2011). Perivascular Adipose Tissue: More than Just Structural Support. Clin. Sci..

[4] Watts S.W., Thompson J.M., Bhattacharya S., Panda V., Terrian L., Contreras A., Nault R. (2024). Integrins Play a Role in Stress Relaxation of Perivascular Adipose Tissue. Pharmacol. Res..

[5] Horimatsu T., Kim H.W., Weintraub N.L. (2017). The Role of Perivascular Adipose Tissue in Non-Atherosclerotic Vascular Disease. Front. Physiol..

[6] Stanek A., Brożyna-Tkaczyk K., Myśliński W. (2021). The Role of Obesity-Induced Perivascular Adipose Tissue (PVAT) Dysfunction in Vascular Homeostasis. Nutrients.

[7] Liu C.-L., Ren J., Wang Y., Zhang X., Sukhova G.K., Liao M., Santos M., Luo S., Yang D., Xia M. (2020). Adipocytes Promote Interleukin-18 Binding to Its Receptors during Abdominal Aortic Aneurysm Formation in Mice. Eur. Heart J..

[8] Angueira A.R., Sakers A.P., Holman C.D., Cheng L., Arbocco M.N., Shamsi F., Lynes M.D., Shrestha R., Okada C., Batmanov K. (2021). Defining the Lineage of Thermogenic Perivascular Adipose Tissue. Nat. Metab..

[9] Chen J.-Y., Wu Y.-P., Li C.-Y., Jheng H.-F., Kao L.-Z., Yang C.-C., Leu S.-Y., Lien I.-C., Weng W.-T., Tai H.-C. (2021). PPARγ Activation Improves the Microenvironment of Perivascular Adipose Tissue and Attenuates Aortic Stiffening in Obesity. J. Biomed. Sci..

[10] Tuttle T., Darios E., Watts S.W., Roccabianca S. (2022). Aortic Stiffness Is Lower When PVAT Is Included: A Novel Ex Vivo Mechanics Study. Am. J. Physiol. Heart Circ. Physiol..

[11] Watts S.W., Flood E.D., Garver H., Fink G.D., Roccabianca S. (2020). A New Function for Perivascular Adipose Tissue (PVAT): Assistance of Arterial Stress Relaxation. Sci. Rep..

[12] Ozen G., Daci A., Norel X., Topal G. (2015). Human Perivascular Adipose Tissue Dysfunction as a Cause of Vascular Disease: Focus on Vascular Tone and Wall Remodeling. Eur. J. Pharmacol..

[13] Villacorta L., Chang L. (2015). The Role of Perivascular Adipose Tissue in Vasoconstriction, Arterial Stiffness, and Aneurysm. Horm. Mol. Biol. Clin. Investig..

[14] Chang L., Garcia-Barrio M.T., Chen Y.E. (2020). Perivascular Adipose Tissue Regulates Vascular Function by Targeting Vascular Smooth Muscle Cells. Arterioscler. Thromb. Vasc. Biol..

[15] Hillock-Watling C., Gotlieb A.I. (2022). The Pathobiology of Perivascular Adipose Tissue (PVAT), the Fourth Layer of the Blood Vessel Wall. Cardiovasc. Pathol..

[16] Ahmed A., Bibi A., Valoti M., Fusi F. (2023). Perivascular Adipose Tissue and Vascular Smooth Muscle Tone: Friends or Foes?. Cells.

[17] Lian X., Gollasch M. (2016). A Clinical Perspective: Contribution of Dysfunctional Perivascular Adipose Tissue (PVAT) to Cardiovascular Risk. Curr. Hypertens. Rep..

[18] Fernández-Alfonso M.S., Gil-Ortega M., García-Prieto C.F., Aranguez I., Ruiz-Gayo M., Somoza B. (2013). Mechanisms of Perivascular Adipose Tissue Dysfunction in Obesity. Int. J. Endocrinol..

[19] Adachi Y., Ueda K., Nomura S., Ito K., Katoh M., Katagiri M., Yamada S., Hashimoto M., Zhai B., Numata G. (2022). Beiging of Perivascular Adipose Tissue Regulates Its Inflammation and Vascular Remodeling. Nat. Commun..

[20] Verhagen S.N., Visseren F.L.J. (2011). Perivascular Adipose Tissue as a Cause of Atherosclerosis. Atherosclerosis.

[21] Zhang S., Jiang J., Luo Y., Liu G., Hu S., Wan S., Luo C., Li H., Li N., da Graça Espírito Santo Vasconcelos J. (2025). Molecular Crosstalk in Perivascular Adipose Tissue: Mechanisms of Inflammation, Metabolic Dysregulation, and Therapeutic Opportunities in Cardiovascular Disease. Front. Cardiovasc. Med..

[22] Gollasch M. (2012). Vasodilator Signals from Perivascular Adipose Tissue. Br. J. Pharmacol..

[23] Antoniades C., Tousoulis D., Vavlukis M., Fleming I., Duncker D.J., Eringa E., Manfrini O., Antonopoulos A.S., Oikonomou E., Padró T. (2023). Perivascular Adipose Tissue as a Source of Therapeutic Targets and Clinical Biomarkers: A Clinical Consensus Statement from the European Society of Cardiology Working Group on Coronary Pathophysiology and Micro-Circulation. Eur. Heart J..

[24] Adachi Y., Ueda K., Takimoto E. (2023). Perivascular Adipose Tissue in Vascular Pathologies—A Novel Therapeutic Target for Atherosclerotic Disease?. Front. Cardiovasc. Med..

[25] Vijay J., Gauthier M.-F., Biswell R.L., Louiselle D.A., Johnston J.J., Cheung W.A., Belden B., Pramatarova A., Biertho L., Gibson M. (2020). Single-Cell Analysis of Human Adipose Tissue Identifies Depot- and Disease-Specific Cell Types. Nat. Metab..

[26] Stuart T., Satija R. (2019). Integrative Single-Cell Analysis. Nat. Rev. Genet..

[27] Fu M., Shu S., Peng Z., Liu X., Chen X., Zeng Z., Yang Y., Cui H., Zhao R., Wang X. (2023). Single-Cell RNA Sequencing of Coronary Perivascular Adipose Tissue From End-Stage Heart Failure Patients Identifies SPP1+ Macrophage Subpopulation as a Target for Alleviating Fibrosis. Arterioscler. Thromb. Vasc. Biol..

[28] Sun F., Li H., Sun D., Fu S., Gu L., Shao X., Wang Q., Dong X., Duan B., Xing F. (2025). Single-Cell Omics: Experimental Workflow, Data Analyses and Applications. Sci. China Life Sci..

[29] Liu X., Zeng Q., Yang H., Li W., Chen Q., Yin K., Pan Z., Wang K., Luo M., Shu C. (2024). Single-Nucleus Multiomic Analyses Identifies Gene Regulatory Dynamics of Phenotypic Modulation in Human Aneurysmal Aortic Root. Adv. Sci..

[30] Li Y., Wang S., Zhang R., Gong Y., Che Y., Li K., Pan Z. (2025). Single-Cell and Spatial Analysis Reveals the Interaction between ITLN1+ Foam Cells and SPP1+ Macrophages in Atherosclerosis. Front. Cardiovasc. Med..

[31] Papalexi E., Mimitou E.P., Butler A.W., Foster S., Bracken B., Mauck W.M., Wessels H.-H., Hao Y., Yeung B.Z., Smibert P. (2021). Characterizing the Molecular Regulation of Inhibitory Immune Checkpoints with Multimodal Single-Cell Screens. Nat. Genet..

[32] Emont M.P., Jacobs C., Essene A.L., Pant D., Tenen D., Colleluori G., Di Vincenzo A., Jørgensen A.M., Dashti H., Stefek A. (2022). A Single-Cell Atlas of Human and Mouse White Adipose Tissue. Nature.

[33] Jaitin D.A., Adlung L., Thaiss C.A., Weiner A., Li B., Descamps H., Lundgren P., Bleriot C., Liu Z., Deczkowska A. (2019). Lipid-Associated Macrophages Control Metabolic Homeostasis in a Trem2-Dependent Manner. Cell.

[34] Hildreth A.D., Ma F., Wong Y.Y., Sun R., Pellegrini M., O’Sullivan T.E. (2021). Single-Cell Sequencing of Human White Adipose Tissue Identifies New Cell States in Health and Obesity. Nat. Immunol..

[35] Li J., Tian Z., Zhang T., Jin J., Zhang X., Xie P., Lin H., Gu J., Wu Y., Wang X. (2024). Single-Cell View and a Novel Protective Macrophage Subset in Perivascular Adipose Tissue in T2DM. Cell. Mol. Biol. Lett..

[36] Thrum S., Sommer M., Raulien N., Gericke M., Massier L., Kovacs P., Krasselt M., Landgraf K., Körner A., Dietrich A. (2022). Macrophages in Obesity Are Characterised by Increased IL-1β Response to Calcium-Sensing Receptor Signals. Int. J. Obes..

[37] Ramirez A.K., Dankel S.N., Rastegarpanah B., Cai W., Xue R., Crovella M., Tseng Y.-H., Kahn C.R., Kasif S. (2020). Single-Cell Transcriptional Networks in Differentiating Preadipocytes Suggest Drivers Associated with Tissue Heterogeneity. Nat. Commun..

[38] Massier L., Jalkanen J., Elmastas M., Zhong J., Wang T., Nono Nankam P.A., Frendo-Cumbo S., Bäckdahl J., Subramanian N., Sekine T. (2023). An Integrated Single Cell and Spatial Transcriptomic Map of Human White Adipose Tissue. Nat. Commun..

[39] Loft A., Emont M.P., Weinstock A., Divoux A., Ghosh A., Wagner A., Hertzel A.V., Maniyadath B., Deplancke B., Liu B. (2025). Towards a Consensus Atlas of Human and Mouse Adipose Tissue at Single-Cell Resolution. Nat. Metab..

[40] Gu W., Nowak W.N., Xie Y., Le Bras A., Hu Y., Deng J., Issa Bhaloo S., Lu Y., Yuan H., Fidanis E. (2019). Single-Cell RNA-Sequencing and Metabolomics Analyses Reveal the Contribution of Perivascular Adipose Tissue Stem Cells to Vascular Remodeling. Arterioscler. Thromb. Vasc. Biol..

[41] Sowka A., Balatskyi V.V., Navrulin V.O., Ntambi J.M., Dobrzyn P. (2025). Stearoyl-CoA Desaturase 1 Regulates Metabolism and Inflammation in Mouse Perivascular Adipose Tissue in Response to a High-Fat Diet. J. Cell. Physiol..

[42] Diaz M.B., Herzig S., Vegiopoulos A. (2014). Thermogenic Adipocytes: From Cells to Physiology and Medicine. Metabolism.

[43] Mu W., Qian S., Song Y., Yang L., Song S., Yang Q., Liu H., Liu Y., Pan D., Tang Y. (2021). BMP4-Mediated Browning of Perivascular Adipose Tissue Governs an Anti-Inflammatory Program and Prevents Atherosclerosis. Redox Biol..

[44] Sakers A., De Siqueira M.K., Seale P., Villanueva C.J. (2022). Adipose Tissue Plasticity in Health and Disease. Cell.

[45] Mikami T., Furuhashi M., Sakai A., Numaguchi R., Harada R., Naraoka S., Kamada T., Higashiura Y., Tanaka M., Ohori S. (2021). Antiatherosclerotic Phenotype of Perivascular Adipose Tissue Surrounding the Saphenous Vein in Coronary Artery Bypass Grafting. J. Am. Heart Assoc..

[46] He W., Yu S., Li J., Li S., Chen Z., Zhang J., Liu Y., Zhou M., Yang T., Cheng W. (2025). From Inflammation to Remodelling: A Novel BASP1+ Monocyte Subset as a Catalyst for Acute Aortic Dissection. J. Adv. Res..

[47] Liu L., Henry J., Liu Y., Jouve C., Hulot J.-S., Georges A., Bouatia-Naji N. (2024). LRP1 Repression by SNAIL Results in ECM Remodeling in Genetic Risk for Vascular Diseases. Circ. Res..

[48] Nosalski R., Mikolajczyk T., Siedlinski M., Saju B., Koziol J., Maffia P., Guzik T.J. (2020). Nox1/4 Inhibition Exacerbates Age Dependent Perivascular Inflammation and Fibrosis in a Model of Spontaneous Hypertension. Pharmacol. Res..

[49] Tai G.-J., Ma Y.-J., Feng J.-L., Li J.-P., Qiu S., Yu Q.-Q., Liu R.-H., Wankumbu S.C., Wang X., Li X.-X. (2024). NLRP3 Inflammasome-Mediated Premature Immunosenescence Drives Diabetic Vascular Aging Dependent on the Induction of Perivascular Adipose Tissue Dysfunction. Cardiovasc. Res..

[50] Shao M., Hepler C., Zhang Q., Shan B., Vishvanath L., Henry G.H., Zhao S., An Y.A., Wu Y., Strand D.W. (2021). Pathologic HIF1α Signaling Drives Adipose Progenitor Dysfunction in Obesity. Cell Stem Cell.

[51] Queiroz M., Sena C.M. (2024). Perivascular Adipose Tissue: A Central Player in the Triad of Diabetes, Obesity, and Cardiovascular Health. Cardiovasc. Diabetol..

[52] Merrick D., Sakers A., Irgebay Z., Okada C., Calvert C., Morley M.P., Percec I., Seale P. (2019). Identification of a Mesenchymal Progenitor Cell Hierarchy in Adipose Tissue. Science.

[53] Chakarov S., Lim H.Y., Tan L., Lim S.Y., See P., Lum J., Zhang X.-M., Foo S., Nakamizo S., Duan K. (2019). Two Distinct Interstitial Macrophage Populations Coexist across Tissues in Specific Subtissular Niches. Science.

[54] Brestoff J.R., Wilen C.B., Moley J.R., Li Y., Zou W., Malvin N.P., Rowen M.N., Saunders B.T., Ma H., Mack M.R. (2021). Intercellular Mitochondria Transfer to Macrophages Regulates White Adipose Tissue Homeostasis and Is Impaired in Obesity. Cell Metab..

[55] Al-Rifai R., Vandestienne M., Lavillegrand J.-R., Mirault T., Cornebise J., Poisson J., Laurans L., Esposito B., James C., Mansier O. (2022). JAK2V617F Mutation Drives Vascular Resident Macrophages toward a Pathogenic Phenotype and Promotes Dissecting Aortic Aneurysm. Nat. Commun..

[56] Guzik T.J., Nosalski R., Maffia P., Drummond G.R. (2024). Immune and Inflammatory Mechanisms in Hypertension. Nat. Rev. Cardiol..

[57] Quelquejay H., Al-Rifai R., Silvestro M., Vandestienne M., Ferreira I., Mirault T., Henrion D., Zhong X., Santos-Zas I., Goudot G. (2024). L-Wnk1 Deletion in Smooth Muscle Cells Causes Aortitis and Inflammatory Shift. Circ. Res..

[58] Xiong J., Li Z., Tang H., Duan Y., Ban X., Xu K., Guo Y., Tu Y. (2023). Bulk and Single-Cell Characterisation of the Immune Heterogeneity of Atherosclerosis Identifies Novel Targets for Immunotherapy. BMC Biol..

[59] Winkels H., Wolf D. (2021). Heterogeneity of T Cells in Atherosclerosis Defined by Single-Cell RNA-Sequencing and Cytometry by Time of Flight. Arterioscler. Thromb. Vasc. Biol..

[60] Yu X., Hu Y., Lim H.Y., Li Z., Jaitin D.A., Yang K., Kong W.T., Xu J., Bejarano D.A., Bied M. (2025). Septal LYVE1+ Macrophages Control Adipocyte Stem Cell Adipogenic Potential. Science.

[61] Di Carlo S.E., Peduto L. (2018). The Perivascular Origin of Pathological Fibroblasts. J. Clin. Investig..

[62] Wirka R.C., Wagh D., Paik D.T., Pjanic M., Nguyen T., Miller C.L., Kundu R., Nagao M., Coller J., Koyano T.K. (2019). Atheroprotective Roles of Smooth Muscle Cell Phenotypic Modulation and the TCF21 Disease Gene as Revealed by Single-Cell Analysis. Nat. Med..

[63] Pang X., He X., Qiu Z., Zhang H., Xie R., Liu Z., Gu Y., Zhao N., Xiang Q., Cui Y. (2023). Targeting Integrin Pathways: Mechanisms and Advances in Therapy. Signal Transduct. Target. Ther..

[64] Rendon C.J., Sempere L., Lauver A., Watts S.W., Contreras G.A. (2024). Anatomical Location, Sex, and Age Modulate Adipocyte Progenitor Populations in Perivascular Adipose Tissues. Front. Physiol..

[65] Xu X., Zhang D.-D., Kong P., Gao Y.-K., Huang X.-F., Song Y., Zhang W.-D., Guo R.-J., Li C.-L., Chen B.-W. (2023). Sox10 Escalates Vascular Inflammation by Mediating Vascular Smooth Muscle Cell Transdifferentiation and Pyroptosis in Neointimal Hyperplasia. Cell Rep..

[66] Dubner A.M., Lu S., Jolly A.J., Strand K.A., Mutryn M.F., Hinthorn T., Noble T., Nemenoff R.A., Moulton K.S., Majesky M.W. (2023). Smooth Muscle–Derived Adventitial Progenitor Cells Direct Atherosclerotic Plaque Composition Complexity in a Klf4-Dependent Manner. JCI Insight.

[67] Chakraborty A., Li Y., Zhang C., Li Y., Rebello K.R., Li S., Xu S., Vasquez H.G., Zhang L., Luo W. (2023). Epigenetic Induction of Smooth Muscle Cell Phenotypic Alterations in Aortic Aneurysms and Dissections. Circulation.

[68] Burl R.B., Ramseyer V.D., Rondini E.A., Pique-Regi R., Lee Y.-H., Granneman J.G. (2018). Deconstructing Adipogenesis Induced by Β3-Adrenergic Receptor Activation with Single-Cell Expression Profiling. Cell Metab..

[69] Huang C., Huang Y., Yao L., Li J., Zhang Z., Huang Z., Chen S., Zhang Y., Wang J., Chen Y. (2023). Thoracic Perivascular Adipose Tissue Inhibits VSMC Apoptosis and Aortic Aneurysm Formation in Mice via the Secretome of Browning Adipocytes. Acta Pharmacol. Sin..

[70] Xu Y., Karbasion N., Toure T.A., Esche J.A., Otani B.K., Snider J.C., Bersi M.R. (2025). Paracrine Signaling by Distinct Adipose Tissue Depots Regulate Fibroblast Mechanobiology and Functional Heterogeneity. Cell. Mol. Bioeng..

[71] Shi H., Goo B., Kim D., Kress T.C., Ogbi M., Mintz J., Wu H., Belin de Chantemèle E.J., Stepp D., Long X. (2023). Perivascular Adipose Tissue Promotes Vascular Dysfunction in Murine Lupus. Front. Immunol..

[72] Sun X., Wu J., Zhang X., Xie C., Wei H., Li P., Yang Y., Yuan H., Cai J., Xiao Q. (2024). Atlas of Cell Repertoire Within Neointimal Lesions Is Metabolically Altered in Hypertensive Rats. Hypertension.

[73] Bäckdahl J., Franzén L., Massier L., Li Q., Jalkanen J., Gao H., Andersson A., Bhalla N., Thorell A., Rydén M. (2021). Spatial Mapping Reveals Human Adipocyte Subpopulations with Distinct Sensitivities to Insulin. Cell Metab..

[74] Pan X., Zhang R., Lu B., Chen S., Chen H., Li M., Qin L., Song Z., Yang Y., Wang Z. (2025). SM22α-Lineage Perivascular Stromal Cells Contribute to Abdominal Aortic Aneurysm. Circ. Res..

[75] Duerre D.J., Galmozzi A. (2022). Deconstructing Adipose Tissue Heterogeneity One Cell at a Time. Front. Endocrinol..

[76] Bersi M.R., Khosravi R., Wujciak A.J., Harrison D.G., Humphrey J.D. (2017). Differential Cell-Matrix Mechanoadaptations and Inflammation Drive Regional Propensities to Aortic Fibrosis, Aneurysm or Dissection in Hypertension. J. R. Soc. Interface.

[77] Kumar R.K., Yang Y., Contreras A.G., Garver H., Bhattacharya S., Fink G.D., Rockwell C.E., Watts S.W. (2021). Phenotypic Changes in T Cell and Macrophage Subtypes in Perivascular Adipose Tissues Precede High-Fat Diet-Induced Hypertension. Front. Physiol..

[78] Lazaro C.M., Freitas I.N., Nunes V.S., Guizoni D.M., Victorio J.A., Oliveira H.C.F., Davel A.P. (2024). Sex-Specific Effects of Cholesteryl Ester Transfer Protein (CETP) on the Perivascular Adipose Tissue. Function.

[79] Tran V., Brettle H., Diep H., Figueiredo Galvao H.B., Fanson K.V., Sobey C.G., Drummond G.R., Vinh A., Jelinic M. (2025). Sex-specific Characterization of Aortic Function and Inflammation in a New Diet-induced Mouse Model of Metabolic Syndrome. FASEB J..

[80] Karbasion N., Xu Y., Snider J.C., Bersi M.R. (2024). Primary Mouse Aortic Smooth Muscle Cells Exhibit Region- and Sex-Dependent Biological Responses In Vitro. J. Biomech. Eng..

[81] Servadei F., Scimeca M., Palumbo V., Oddi F.M., Bonfiglio R., Giacobbi E., Menghini R., Casagrande V., Cardellini M., Martelli E. (2025). Aging and Sex Modify the Risk of Carotid Plaque Thrombosis Related to Dyslipidemic Profile. Stroke.

[82] Wang Z., Lu H., Garcia-Barrio M., Guo Y., Zhang J., Chen Y.E., Chang L. (2022). RNA Sequencing Reveals Perivascular Adipose Tissue Plasticity in Response to Angiotensin II. Pharmacol. Res..

[83] Shi N., Xia J., Wang C., Zhou J., Huang J., Hu M., Liao J. (2022). Aerobic Exercise Prevents Arterial Stiffness and Attenuates Hyperexcitation of Sympathetic Nerves in Perivascular Adipose Tissue of Mice after Transverse Aortic Constriction. Int. J. Mol. Sci..

[84] Zhang Z.-B., Cheng Y.-W., Xu L., Li J.-Q., Pan X., Zhu M., Chen X.-H., Sun A.-J., Lin J.-R., Gao P.-J. (2024). Activation of Β3-Adrenergic Receptor by Mirabegron Prevents Aortic Dissection/Aneurysm by Promoting Lymphangiogenesis in Perivascular Adipose Tissue. Cardiovasc. Res..

[85] Rajbhandari P., Arneson D., Hart S.K., Ahn I.S., Diamante G., Santos L.C., Zaghari N., Feng A.-C., Thomas B.J., Vergnes L. (2019). Single Cell Analysis Reveals Immune Cell–Adipocyte Crosstalk Regulating the Transcription of Thermogenic Adipocytes. eLife.

[86] Liu X., Jiang X., Hu J., Ding M., Lee S.K., Korivi M., Qian Y., Li T., Wang L., Li W. (2024). Exercise Attenuates High-Fat Diet-Induced PVAT Dysfunction through Improved Inflammatory Response and BMP4-Regulated Adipose Tissue Browning. Front. Nutr..

[87] Pan X.-X., Ruan C.-C., Liu X.-Y., Kong L.-R., Ma Y., Wu Q.-H., Li H.-Q., Sun Y.-J., Chen A.-Q., Zhao Q. (2019). Perivascular Adipose Tissue-Derived Stromal Cells Contribute to Vascular Remodeling during Aging. Aging Cell.

[88] Arner E., Mejhert N., Kulyté A., Balwierz P.J., Pachkov M., Cormont M., Lorente-Cebrián S., Ehrlund A., Laurencikiene J., Hedén P. (2012). Adipose Tissue MicroRNAs as Regulators of CCL2 Production in Human Obesity. Diabetes.

[89] Valladares A., Roncero C., Benito M., Porras A. (2001). TNF-α Inhibits UCP-1 Expression in Brown Adipocytes via ERKs: Opposite Effect of p38MAPK. FEBS Lett..

[90] Kwartler C.S., Pedroza A.J., Kaw A., Guan P., Ma S., Duan X., Kernell C., Wang C., Pinelo J.E.E., Bowen M.S.B. (2023). Nuclear Smooth Muscle α-Actin Participates in Vascular Smooth Muscle Cell Differentiation. Nat. Cardiovasc. Res..

[91] Callow B., He X., Juriga N., Mangum K.D., Joshi A., Xing X., Obi A., Chattopadhyay A., Milewicz D.M., O’Riordan M.X. Inhibition of Vascular Smooth Muscle Cell PERK/ATF4 ER Stress Signaling Protects Against Abdominal Aortic Aneurysms. https://insight.jci.org/articles/view/183959/pdf.

[92] Zhang R., Gao Y., Zhao X., Gao M., Wu Y., Han Y., Qiao Y., Luo Z., Yang L., Chen J. (2018). FSP1-Positive Fibroblasts Are Adipogenic Niche and Regulate Adipose Homeostasis. PLoS Biol..

[93] Kesharwani D., Brown A.C. (2024). Navigating the Adipocyte Precursor Niche: Cell-Cell Interactions, Regulatory Mechanisms and Implications for Adipose Tissue Homeostasis. J. Cell. Signal..

[94] Dong H., Sun W., Shen Y., Baláz M., Balázová L., Ding L., Löffler M., Hamilton B., Klöting N., Blüher M. (2022). Identification of a Regulatory Pathway Inhibiting Adipogenesis via RSPO2. Nat. Metab..

[95] Nahmgoong H., Kim J.B. (2025). Mapping Macrophage-Fibroblast Interactions in Adipose Tissue Fibrosis. Diabetes.

[96] Wu X., Peng J., Jin C. (2025). Deciphering Macrophage-Fibroblast Cross Talk in Obesity-Induced Adipose Tissue Fibrosis: Insights From Single-Cell and Spatial Transcriptomics. Diabetes.

[97] Raddatz M.A., Huffstater T.M., Bersi M.R., Reinfeld B.I., Madden M.Z., Booton S.E., Rathmell W.K., Rathmell J.C., Lindman B.R., Madhur M.S. (2020). Macrophages Promote Aortic Valve Cell Calcification and Alter STAT3 Splicing. Arterioscler. Thromb. Vasc. Biol..

[98] Nanduri R., Furusawa T., Lobanov A., He B., Xie C., Dadkhah K., Kelly M.C., Gavrilova O., Gonzalez F.J., Bustin M. (2022). Epigenetic Regulation of White Adipose Tissue Plasticity and Energy Metabolism by Nucleosome Binding HMGN Proteins. Nat. Commun..

[99] Park Y.J., Lee S., Lim S., Nahmgoong H., Ji Y., Huh J.Y., Alfadda A.A., Kim S., Kim J.B. (2021). DNMT1 Maintains Metabolic Fitness of Adipocytes through Acting as an Epigenetic Safeguard of Mitochondrial Dynamics. Proc. Natl. Acad. Sci. USA.

[100] Wang J., Tontonoz P. (2017). Pioneering EBF2 Remodels the Brown Fat Chromatin Landscape. Genes Dev..

[101] Hiraike Y., Waki H., Yu J., Nakamura M., Miyake K., Nagano G., Nakaki R., Suzuki K., Kobayashi H., Yamamoto S. (2017). NFIA Co-Localizes with PPARγ and Transcriptionally Controls the Brown Fat Gene Program. Nat. Cell Biol..

[102] Jiang N., Yang M., Han Y., Zhao H., Sun L. (2022). PRDM16 Regulating Adipocyte Transformation and Thermogenesis: A Promising Therapeutic Target for Obesity and Diabetes. Front. Pharmacol..

[103] Lee S., Benvie A.M., Park H.G., Spektor R., Harlan B., Brenna J.T., Berry D.C., Soloway P.D. (2022). Remodeling of Gene Regulatory Networks Underlying Thermogenic Stimuli-Induced Adipose Beiging. Commun. Biol..

[104] Wang Z., Cheng J., Wang Y., Yuan H., Bi S., Wang S., Hou Y., Zhang X., Xu B., Wang Z. (2024). Macrophage ILF3 Promotes Abdominal Aortic Aneurysm by Inducing Inflammatory Imbalance in Male Mice. Nat. Commun..

[105] Klingelhuber F., Frendo-Cumbo S., Omar-Hmeadi M., Massier L., Kakimoto P., Taylor A.J., Couchet M., Ribicic S., Wabitsch M., Messias A.C. (2024). A Spatiotemporal Proteomic Map of Human Adipogenesis. Nat. Metab..

[106] Jin S., Guerrero-Juarez C.F., Zhang L., Chang I., Ramos R., Kuan C.-H., Myung P., Plikus M.V., Nie Q. (2021). Inference and Analysis of Cell-Cell Communication Using CellChat. Nat. Commun..

[107] Browaeys R., Saelens W., Saeys Y. (2020). NicheNet: Modeling Intercellular Communication by Linking Ligands to Target Genes. Nat. Methods.

[108] Britton K.A., Fox C.S. (2011). Perivascular Adipose Tissue and Vascular Disease. Clin. Lipidol..

[109] Brückner A., Brandtner A., Rieck S., Matthey M., Geisen C., Fels B., Stei M., Kusche-Vihrog K., Fleischmann B.K., Wenzel D. (2024). Site-Specific Genetic and Functional Signatures of Aortic Endothelial Cells at Aneurysm Predilection Sites in Healthy and AngII ApoE−/− Mice. Angiogenesis.

[110] Maniyadath B., Zhang Q., Gupta R.K., Mandrup S. (2023). Adipose Tissue at Single-Cell Resolution. Cell Metab..

[111] Chen J.-Y., Zhu X.-L., Liu W.-H., Xie Y., Zhang H.-F., Wang X., Ying R., Chen Z.-T., Wu M.-X., Qiu Q. (2020). C-Reactive Protein Derived from Perivascular Adipose Tissue Accelerates Injury-Induced Neointimal Hyperplasia. J. Transl. Med..

[112] Kim E.N., Seok H.Y., Lim J.S., Koh J., Bae J.M., Kim C.J., Ryu G.-H., Ok Y.J., Choi J.-S., Cho C.-H. (2024). CRP Deposition in Human Abdominal Aortic Aneurysm Is Associated with Transcriptome Alterations toward Aneurysmal Pathogenesis: Insights from in Situ Spatial Whole Transcriptomic Analysis. Front. Immunol..

[113] Chen M., Zhu J., Luo H., Mu W., Guo L. (2024). The Journey towards Physiology and Pathology: Tracing the Path of Neuregulin 4. Genes Dis..

[114] Henriques F., Bedard A.H., Guilherme A., Kelly M., Chi J., Zhang P., Lifshitz L.M., Bellvé K., Rowland L.A., Yenilmez B. (2020). Single-Cell RNA Profiling Reveals Adipocyte to Macrophage Signaling Sufficient to Enhance Thermogenesis. Cell Rep..

[115] Schumacher M.A., Dennis I.C., Liu C.Y., Robinson C., Shang J., Bernard J.K., Washington M.K., Polk D.B., Frey M.R. (2021). NRG4-ErbB4 Signaling Represses Proinflammatory Macrophage Activity. Am. J. Physiol.-Gastrointest. Liver Physiol..

[116] Korhonen E.A., Murtomäki A., Jha S.K., Anisimov A., Pink A., Zhang Y., Stritt S., Liaqat I., Stanczuk L., Alderfer L. (2022). Lymphangiogenesis Requires Ang2/Tie/PI3K Signaling for VEGFR3 Cell-Surface Expression. J. Clin. Investig..

[117] Sárvári A.K., Hauwaert E.L.V., Markussen L.K., Gammelmark E., Marcher A.-B., Ebbesen M.F., Nielsen R., Brewer J.R., Madsen J.G.S., Mandrup S. (2021). Plasticity of Epididymal Adipose Tissue in Response to Diet-Induced Obesity at Single-Nucleus Resolution. Cell Metab..

[118] Nitti M.D., Hespe G.E., Kataru R.P., García Nores G.D., Savetsky I.L., Torrisi J.S., Gardenier J.C., Dannenberg A.J., Mehrara B.J. (2016). Obesity-induced Lymphatic Dysfunction Is Reversible with Weight Loss. J. Physiol..

[119] Cao E., Watt M.J., Nowell C.J., Quach T., Simpson J.S., De Melo Ferreira V., Agarwal S., Chu H., Srivastava A., Anderson D. (2021). Mesenteric Lymphatic Dysfunction Promotes Insulin Resistance and Represents a Potential Treatment Target in Obesity. Nat. Metab..

[120] Yeo K.P., Lim H.Y., Angeli V. (2021). Leukocyte Trafficking via Lymphatic Vessels in Atherosclerosis. Cells.

[121] Oliver G., Kipnis J., Randolph G.J., Harvey N.L. (2020). The Lymphatic Vasculature in the 21st Century: Novel Functional Roles in Homeostasis and Disease. Cell.

[122] AlZaim I., de Rooij L.P.M.H., Sheikh B.N., Börgeson E., Kalucka J. (2023). The Evolving Functions of the Vasculature in Regulating Adipose Tissue Biology in Health and Obesity. Nat. Rev. Endocrinol..

[123] Kral M., van der Vorst E.P.C., Weber C., Döring Y. (2024). (Multi-) Omics Studies of ILC2s in Inflammation and Metabolic Diseases. Front. Cell Dev. Biol..

[124] Misawa T., Wagner M., Koyasu S. (2022). ILC2s and Adipose Tissue Homeostasis: Progress to Date and the Road Ahead. Front. Immunol..

[125] Huang Y., Mao K., Chen X., Sun M., Kawabe T., Li W., Usher N., Zhu J., Urban J.F., Paul W.E. (2018). S1P-Dependent Interorgan Trafficking of Group 2 Innate Lymphoid Cells Supports Host Defense. Science.

[126] Zhao Y., Xiong W., Li C., Zhao R., Lu H., Song S., Zhou Y., Hu Y., Shi B., Ge J. (2023). Hypoxia-Induced Signaling in the Cardiovascular System: Pathogenesis and Therapeutic Targets. Signal Transduct. Target. Ther..

[127] Grunewald M., Kumar S., Sharife H., Volinsky E., Gileles-Hillel A., Licht T., Permyakova A., Hinden L., Azar S., Friedmann Y. (2021). Counteracting Age-Related VEGF Signaling Insufficiency Promotes Healthy Aging and Extends Life Span. Science.

[128] Auvinen J., Tapio J., Karhunen V., Kettunen J., Serpi R., Dimova E.Y., Gill D., Soininen P., Tammelin T., Mykkänen J. (2021). Systematic Evaluation of the Association between Hemoglobin Levels and Metabolic Profile Implicates Beneficial Effects of Hypoxia. Sci. Adv..

[129] Chen H.-J., Sévin D.C., Griffith G.R., Vappiani J., Booty L.M., van Roomen C.P.A.A., Kuiper J., den Dunnen J., de Jonge W.J., Prinjha R.K. (2024). Integrated Metabolic-Transcriptomic Network Identifies Immunometabolic Modulations in Human Macrophages. Cell Rep..

[130] Plissonneau C., Santosa S. (2024). Regional Primary Preadipocyte Characteristics in Humans with Obesity and Type 2 Diabetes Mellitus. Heliyon.

[131] Reggio A., Fuoco C., Deodati R., Palma A. (2025). SPP1 Macrophages across Diseases: A Call for Reclassification?. FASEB J..

[132] Liu J., Zhang Y., Tian Y., Huang W., Tong N., Fu X. (2022). Integrative Biology of Extracellular Vesicles in Diabetes Mellitus and Diabetic Complications. Theranostics.

[133] Fu E., Pan K., Li Z. (2024). Engineering Extracellular Vesicles for Targeted Therapeutics in Cardiovascular Disease. Front. Cardiovasc. Med..

[134] Pan Y., Hui X., Hoo R.L.C., Ye D., Chan C.Y.C., Feng T., Wang Y., Lam K.S.L., Xu A. (2019). Adipocyte-Secreted Exosomal microRNA-34a Inhibits M2 Macrophage Polarization to Promote Obesity-Induced Adipose Inflammation. J. Clin. Investig..

[135] Liu S., Yang Y., Jiang S., Xu H., Tang N., Lobo A., Zhang R., Liu S., Yu T., Xin H. (2019). MiR-378a-5p Regulates Proliferation and Migration in Vascular Smooth Muscle Cell by Targeting CDK1. Front. Genet..

[136] Sanz-Gómez M., Manzano-Lista F.J., Vega-Martín E., González-Moreno D., Alcalá M., Gil-Ortega M., Somoza B., Pizzamiglio C., Ruilope L.M., Aránguez I. (2023). Finerenone Protects against Progression of Kidney and Cardiovascular Damage in a Model of Type 1 Diabetes through Modulation of Proinflammatory and Osteogenic Factors. Biomed. Pharmacother..

[137] Bakris G.L., Agarwal R., Anker S.D., Pitt B., Ruilope L.M., Rossing P., Kolkhof P., Nowack C., Schloemer P., Joseph A. (2020). Effect of Finerenone on Chronic Kidney Disease Outcomes in Type 2 Diabetes. N. Engl. J. Med..

[138] Filippatos G., Anker S.D., Agarwal R., Pitt B., Ruilope L.M., Rossing P., Kolkhof P., Schloemer P., Tornus I., Joseph A. (2021). Finerenone and Cardiovascular Outcomes in Patients With Chronic Kidney Disease and Type 2 Diabetes. Circulation.

[139] Pitt B., Filippatos G., Agarwal R., Anker S.D., Bakris G.L., Rossing P., Joseph A., Kolkhof P., Nowack C., Schloemer P. (2021). Cardiovascular Events with Finerenone in Kidney Disease and Type 2 Diabetes. N. Engl. J. Med..

[140] Azul L., Leandro A., Seiça R., Sena C.M. (2024). Propagermanium as a Novel Therapeutic Approach for the Treatment of Endothelial Dysfunction in Type 2 Diabetes. Int. J. Mol. Sci..

[141] Ridker P.M., Everett B.M., Thuren T., MacFadyen J.G., Chang W.H., Ballantyne C., Fonseca F., Nicolau J., Koenig W., Anker S.D. (2017). Antiinflammatory Therapy with Canakinumab for Atherosclerotic Disease. N. Engl. J. Med..

[142] Garcia B., Francois-Vaughan H., Onikoyi O., Kostadinov S., Paepe M.E.D., Gruppuso P.A., Sanders J.A. (2014). Xenotransplantation of Human Fetal Adipose Tissue: A Model of in Vivo Adipose Tissue Expansion and Adipogenesis. J. Lipid Res..

[143] Ablamunits V., Klebanov S., Giese S.Y., Herold K.C. (2012). Functional Human to Mouse Adipose Tissue Xenotransplantation. J. Endocrinol..

[144] Adipocyte-Derived Extracellular Vesicles Increase Insulin Secretion through Transport of Insulinotropic Protein Cargo|Nature Communications. https://www.nature.com/articles/s41467-023-36148-1.

[145] Mishra S., Kumar A., Kim S., Su Y., Singh S., Sharma M., Almousa S., Rather H.A., Jain H., Lee J. (2023). A Liquid Biopsy-Based Approach to Isolate and Characterize Adipose Tissue-Derived Extracellular Vesicles from Blood. ACS Nano.

[146] Zhang Z., Ling T., Ding Q., Zhu F., Cheng X., Li X., Ma T., Meng Q. (2025). GlycoRNA-Rich, Neutrophil Membrane-Coated, siMT1-Loaded Nanoparticles Mitigate Abdominal Aortic Aneurysm Progression by Inhibiting the Formation of Neutrophil Extracellular Traps. Mater. Today Bio.

[147] Klüner L.V., Oikonomou E.K., Antoniades C. (2021). Assessing Cardiovascular Risk by Using the Fat Attenuation Index in Coronary CT Angiography. Radiol. Cardiothorac. Imaging.

[148] Chan K., Wahome E., Tsiachristas A., Antonopoulos A.S., Patel P., Lyasheva M., Kingham L., West H., Oikonomou E.K., Volpe L. (2024). Inflammatory Risk and Cardiovascular Events in Patients without Obstructive Coronary Artery Disease: The ORFAN Multicentre, Longitudinal Cohort Study. Lancet.

[149] Oikonomou E.K., Antonopoulos A.S., Schottlander D., Marwan M., Mathers C., Tomlins P., Siddique M., Klüner L.V., Shirodaria C., Mavrogiannis M.C. (2021). Standardized Measurement of Coronary Inflammation Using Cardiovascular Computed Tomography: Integration in Clinical Care as a Prognostic Medical Device. Cardiovasc. Res..

[150] Walker B.L., Cang Z., Ren H., Bourgain-Chang E., Nie Q. (2022). Deciphering Tissue Structure and Function Using Spatial Transcriptomics. Commun. Biol..

[151] González-Amor M., Vila-Bedmar R., Rodrigues-Díez R., Moreno-Carriles R., Arcones A.C., Cruces-Sande M., Salaices M., Mayor F., Briones A.M., Murga C. (2020). Myeloid GRK2 Regulates Obesity-Induced Endothelial Dysfunction by Modulating Inflammatory Responses in Perivascular Adipose Tissue. Antioxidants.

[152] Norreen-Thorsen M., Struck E.C., Öling S., Zwahlen M., Von Feilitzen K., Odeberg J., Lindskog C., Pontén F., Uhlén M., Dusart P.J. (2022). A Human Adipose Tissue Cell-Type Transcriptome Atlas. Cell Rep..

[153] Noutahi E., Hartford J., Tossou P., Whitfield S., Denton A.K., Wognum C., Ulicna K., Craig M., Hsu J., Cuccarese M. (2025). Virtual Cells: Predict, Explain, Discover. arXiv.

[154] Zhang K., Yang X., Wang Y., Yu Y., Huang N., Li G., Li X., Wu J.C., Yang S. (2025). Artificial Intelligence in Drug Development. Nat. Med..

[155] Wang G., Liu T., Zhao J., Cheng Y., Zhao H. (2024). Modeling and Predicting Single-Cell Multi-Gene Perturbation Responses with scLAMBDA. bioRxiv.

